# Extensive Transcriptional Regulation of Chromatin Modifiers during Human Neurodevelopment

**DOI:** 10.1371/journal.pone.0036708

**Published:** 2012-05-09

**Authors:** Matthias K. Weng, Bastian Zimmer, Dominik Pöltl, Marc P. Broeg, Violeta Ivanova, John A. Gaspar, Agapios Sachinidis, Ullrich Wüllner, Tanja Waldmann, Marcel Leist

**Affiliations:** 1 Doerenkamp-Zbinden Department of In Vitro Toxicology and Biomedicine, University of Konstanz, Konstanz, Germany; 2 Konstanz Research School Chemical Biology, University of Konstanz, Konstanz, Germany; 3 Department of Computer Graphics and Media Design, University of Konstanz, Konstanz, Germany; 4 Department of Bioinformatics and Information Mining, University of Konstanz, Konstanz, Germany; 5 Graduate School for Computer and Information Science, University of Konstanz, Konstanz, Germany; 6 Institute of Neurophysiology, University of Cologne, Cologne, Germany; 7 Department of Neurology, Bonn University Hospital, Bonn, Germany; Instituto de Medicina Molecular, Portugal

## Abstract

Epigenetic changes, including histone modifications or chromatin remodeling are regulated by a large number of human genes. We developed a strategy to study the coordinate regulation of such genes, and to compare different cell populations or tissues. A set of 150 genes, comprising different classes of epigenetic modifiers was compiled. This new tool was used initially to characterize changes during the differentiation of human embryonic stem cells (hESC) to central nervous system neuroectoderm progenitors (NEP). qPCR analysis showed that more than 60% of the examined transcripts were regulated, and >10% of them had a >5-fold increased expression. For comparison, we differentiated hESC to neural crest progenitors (NCP), a distinct peripheral nervous system progenitor population. Some epigenetic modifiers were regulated into the same direction in NEP and NCP, but also distinct differences were observed. For instance, the remodeling ATPase SMARCA2 was up-regulated >30-fold in NCP, while it remained unchanged in NEP; up-regulation of the ATP-dependent chromatin remodeler CHD7 was increased in NEP, while it was down-regulated in NCP. To compare the neural precursor profiles with those of mature neurons, we analyzed the epigenetic modifiers in human cortical tissue. This resulted in the identification of 30 regulations shared between all cell types, such as the histone methyltransferase SETD7. We also identified new markers for post-mitotic neurons, like the arginine methyl transferase PRMT8 and the methyl transferase EZH1. Our findings suggest a hitherto unexpected extent of regulation, and a cell type-dependent specificity of epigenetic modifiers in neurodifferentiation.

## Introduction

Chromatin structure is a major determinant of gene expression. The transcriptionally active “open” euchromatin and the silenced “closed” heterochromatin are two major structural variants. Increasing evidence points to intermediate forms which undergo dynamic changes especially during development [1]. The mechanisms determining access of transcription factors to their regulatory sequences include DNA methylation [2], post-translational modifications (PTM) of histones [3] and chromatin remodeling [4]. Histones, the most abundant chromatin proteins, are the building blocks of the nucleosomes. The latter consist of DNA wrapped around an octameric histone core [5]. Modifications of histones, and binding of further chromatin-associated proteins act in concert with DNA methylation to regulate access to the genetic information [6], [7], [8]. These control mechanisms that are independent of the primary DNA sequence are jointly termed “epigenetics”.

The enzymes catalyzing PTM of histones are classified as “writers”. They promote acetylation, methylation and ubiquitination of specific lysine residues or the phosphorylation of serines and threonines. Enzymes removing these histone marks, such as KDM6B, act as “erasers” [3]. Different binding proteins, such as the heterochromatin protein-1 (HP1) are recruited by specific histone modifications [6]. These “readers” translate the code of the histone marks into structural changes of the chromatin. For many marks, e.g. the methylation of histone H3 at lysine 4 (H3K4me), multiple writers, readers and erasers are known [3]. Additional important players in chromatin dynamics are the multi-subunit chromatin remodeling complexes, such as the BAF complex, which can alter chromatin structure in an ATP-dependent manner [4].

Although the first genome-wide maps of histone modifications have been assembled for different human cell types [9], [10], [11], only scarce information is available on the expression, function and role of the different epigenetic regulators and modifiers during human neurodevelopmental processes. Most information on neuronal chromatin modifiers has been derived from neuroblastoma cell lines [12], and data on non-transformed cells are mainly of rodent origin. Epigenetic mechanisms have been studied during mouse development, both *in vivo* [13] and in differentiating murine embryonic stem cells (ESC) *in vitro* [14], [15]. These studies revealed interesting information on the lineage-specific expression of some chromatin remodelers such as the BAF complex. Its neural progenitor stage-specific subunits (BAF45A, BAF53A) are exchanged for the neuron-specific (BAF45C, BAF53B) counterparts [16].

Differentiating hESC represent an interesting new model system to study early neural development, and allow the generation of pure populations of early neuroectoderm CNS progenitors (NEP) [17] and neural crest cells, progenitors of the PNS (NCP) [18], [19]. In these systems, the gene expression changes of chromatin modifiers occurring at different stages of development can be characterized, and differences between PNS and CNS progenitors may be identified.

In the present study we used bioinformatic methods and literature evaluation to select genes covering the major families of epigenetic modifiers and human chromatin associated proteins. Then, we assembled a representative list of 150 candidates to be studied. Their expression was quantified in human brain as well as in two tightly-controlled differentiation models, resulting in NEP and NCP cells, in order to investigate potential lineage- and stage-specific gene expression patterns of epigenetic regulators, and to validate the usefulness of the gene set for the characterization of tissue-specific profiles.

## Materials and Methods

### Cultivation of the hESC line H9

H9 cells were cultivated in HES medium (DMEM/F12, 20% knock-out serum replacement (KSR), 1x GlutaMax, 100 µM MEM NEAA, 90 µM beta-mercaptoethanol and 10 ng/ml FGF-2) on feeder cells (mouse embryonic fibroblasts (MEF)) at 37°C and 5% CO_2_. Every five to six days the hESC colonies were passaged by treatment with dispase for 9 min, and plated on MEF that had been seeded one day before. Medium was changed daily. The hESC WA09 (H9) was obtained from the Wisconsin International Stem Cell Bank (WISC Bank). Import of the cells and all experiments were carried out according to German legislation under the license number 1710-79-1-4-27 of the Robert-Koch Institute.

### Differentiation of H9 to NEP

Differentiation towards NEP was performed as described before [17], with the following minor change: instead of high noggin concentrations, we used a combination of noggin and dorsomorphin [20]. From day 0 on, cells were grown in media containing 35 ng/ml noggin *plus* 600 nM dorsomorphin in addition to the earlier described constituent SB 431542 (10 µM) [17]. On day 10 of differentiation, cells were quality-controlled by immunostaining. Populations containing >90% PAX6-positive cells were harvested for mRNA preparation.

### Differentiation of H9 to NCP

The hESC line H9 was differentiated into NCP exactly as described earlier by Lee et al. [18], [19]. Briefly, cells were grown on the stromal feeder cell line MS5 [56] in KSR medium without FGF-2. After 3 days of differentiation, 500 ng/ml noggin were added to the culture. After 4 additional days of differentiation, KSR medium was additionally supplemented with sonic hedgehog (Shh; 200 ng/ml) and fibroblast growth factor 8 (FGF-8; 100 ng/ml). After 5 more days, medium was changed to DMEM/F12 medium containing N2 constituents [19] (N2 medium) supplemented with Shh, FGF-8, brain-derived neurotrophic factor (BDNF, 20 ng/ml) and ascorbic acid (200 µM). On day 21, rosettes were manually picked and transferred to poly-L-ornithine (10 µg/ml), laminin (1 µg/ml) and fibronectin (1 µg/ml) coated dishes. Cells were grown in N2 medium, supplemented with BDNF, Shh, FGF-8 and ascorbic acid for 7 additional days. On day 28, cells were FACS-purified, using positive sorting for cells expressing HNK1 and p75. The population was always >90% double-positive for the two NCP markers.

### Human brain samples

The cortex of three neurologically healthy control individuals (mean age 75±10 years), provided by the German Brain-Net (Munich, Germany) was used for analysis. Post-mortem cortex samples had been obtain after written consent of the subjects and the next of kin, in adherence to the guidelines laid down in the Declaration of Helsinki on human research ethics. The use of the material for this study was specifically approved by the IRB of the University of Konstanz. RNA was extracted from frozen tissue and converted to cDNA as described below.

### Immunostaining

Cells were grown and differentiated on glass cover slips and fixed with PBS, 4% para-formaldehyde, 2% sucrose for 15 minutes. After permeabilization with 0.2% Triton-x-100 in PBS for 7 minutes, the cells were blocked for one hour in blocking solution (PBS, 1% BSA, 0.1% Triton-x-100). Primary and secondary antibodies were diluted in blocking solution and incubated for one hour each. DNA was stained with Hoechst-33342 and mounted with Fluorsave reagent (Calbiochem).

Images were taken with an IX81 inverted microscope (Olympus, Hamburg, Germany) equipped with a 40x air objective and processed using Cell^P^ imaging software (Olympus). For confocal microscopy, cover slips were mounted using Vectashield (containing DAPI), images were taken with a Zeiss LSM 510Meta confocal microscope equipped with a Plan Apochromat 63x, NA 1.4 oil DIC lens. Images were processed, using Adobe Photoshop CS2, and antigens are displayed in false colors as indicated by the antigen label in the figures.

### Western blot analysis

Undifferentiated hESC and differentiated NEP were lysed in 2% SDS, followed by sonification to fragment genomic DNA. Samples were separated on 18.7% gels by SDS-PAGE. Proteins were transferred onto nitrocellulose membranes (Amersham; Buckinghamshire, UK) using a BioRad WetBlot device. After 1 h blocking with 4% BSA in TBS containing 0.5% Tween-40 (TBS-T), membranes were incubated with primary antibodies over night at 4°C. Following washing steps with TBS-T, membranes were incubated with anti-rabbit-HRP (1∶10000, Jackson Immuno Research) for 1 h at RT. For visualization, ECL Western blotting substrate (Pierce) was used. Used primary antibodies are listed in Figure S1. Loading of equal amounts of histones was controlled by staining for total H3.

### Chromatin immuno precipitation

The chromatin immunoprecipitation assay on native chromatin (N-ChIP) was performed according to the detailed protocol of Umlauf and colleagues [21].

Briefly, for ChIP analysis either undifferentiated hESC or cells on day 10 of differentiation were used. During MNase digestion, the nuclei of 5×10^6^ cells were resuspended in 250 µl of digestion buffer and treated for 12–14 min with 80 units of MNase at 25°C. For immunoprecipitation we used chromatin fractions consisting of 1 to 5 nucleosomes and precipitated them with 2 µl of H3K4me3 (Millipore #17-614) and H3K27me3 (Active Motif #39535), respectively. Data are presented as enrichment relative to unspecific control.

### Reverse transcription and quantitative qPCR

For reverse transcription quantitative PCR (qPCR) analysis, RNA was extracted with the RNeasy mini Kit (Qiagen, Hilden, Germany). The cDNA synthesis was performed using the cDNA synthesis kit from SABiosciences or from Invitrogen. Primers were designed (see Figure S1) according to the following requirements: exon-spanning primers were designed manually and optimized for melting temperature and primer dimerization by using the Oligoanalyzer tool from IDT (http://eu.idtdna.com/analyzer/applications-/oligoanalyzer/). Afterwards they were tested for nonspecific amplification products through melt curve analysis and agarose gel electrophoresis. All qPCRs were run in a Biorad Light Cycler (Biorad, München, Germany) using the following settings: 1× (10 min 95°C), 40× (30 sec 95°C, 30 sec 60°C, 45 sec 72°C) or the settings described for the RT^2^ Profiler^TM^ PCR arrays by SABiosciences. A large part of the primer sets used here is available on pre-assembled plates as RT^2^ Profiler^TM^ PCR Arrays (“Human Neurogenesis and Neural Stem Cell” (PAHS-404A), “Human Epigenetic Chromatin Modification Enzymes” (PAHS-085A), and “Human Epigenetic Chromatin Remodeling Factors” (PAHS-086A), all from SABiosciences, Frederick, MD, USA). Data in figures are shown as means ± SEM of three independent differentiations. For statistical analysis, we used the data calculated with the ΔCt method and performed two-tailed t-test with Welch correction for different variances between hESC, NEP, NCP or cortex. In a second step we corrected the p-values for multiplicity via Benjamini-Hochberg FDR (false discovery rate)-correction. For p-values <0.05, the regulation levels were assumed to be significant and marked with an asterisk.

### Normalization of qPCR data for cell type comparisons

The threshold cycle values (Ct) determined with the iQ5 optical system software (Bio-Rad) were exported to Microsoft Excel for further analysis. To evaluate the stability of the 5 reference genes present on the array, the *geNorm* macro for Microsoft Excel was used [22]. Gene expression stability (M) was calculated with *geNorm*, and the genes were ranked from best to worst, based on the M value. *geNorm* determines the individual stability of a gene within a pool of genes, and calculates the stability according to the similarity of their expression profile by pair-wise comparison, using the geometric mean as a normalizing factor. The gene with the highest M, i.e. the least stable gene, is then excluded in a stepwise fashion until the most stable genes are determined. This way we ended up with four reference genes (Actb/HPRT1/RPL13A/GAPDH) that showed M-values ranging from 0.41 to 0.59 depending on the data set analyzed. Calculation of the relative expression values (fold change or (2^−(ΔΔCt)^)) of all genes was performed using the comparative Ct method [23], [24].

### Affymetrix gene chip analysis

hESC were differentiated either towards NEP or NCP as described above, and samples from approximately 5x10^6^ cells (hESC, NEP, NCP) were collected using RNAprotect reagent from Qiagen. The RNA was quantified using a NanoDrop N-1000 spectrophotometer (NanoDrop, Wilmington, DE, USA), and the integrity of RNA was confirmed with a standard sense automated gel electrophoresis system (Experion, Bio-Rad, Hercules, CA, USA). The samples were taken for transcriptional profiling when the RNA quality indicator (RQI) number was >8. First-strand cDNA was synthesized from 100 ng total RNA using an oligo-dT primer with an attached T7 promoter sequence and then, the complementary second strand was made. The double-stranded cDNA molecule was used for in vitro transcription (IVT, standard Affymetrix procedure) using Genechip 3′ IVT Express Kit. As the aRNA (amplified RNA, also commonly referred to as cRNA) is being made, a biotinylated nucleotide analog is incorporated and serves as a label for the message. After amplification, aRNA was purified with magnetic beads, and 15 μg of aRNA were fragmented with fragmentation buffer as per the manufacturer's instructions. Then, 12.5 μg fragmented aRNA were hybridized with Affymetrix Human Genome U133 plus 2.0 arrays as per the manufacturer's instructions. The chips were placed in a GeneChip Hybridization Oven-645 for 16 h at 60 rpm and 45°C. For staining and washing, Affymetrix HWS kits were used on a Genechip Fluidics Station-450. For scanning, the Affymetrix Gene-Chip Scanner-3000-7G was used, and the image and quality control assessments were performed with Affymetrix GCOS software. All reagents and instruments were acquired from Affymetrix (Affymetrix, Santa Clara, CA, USA). The generated CEL files were taken for further statistical analysis. The authors declare that microarray data are produced according to MIAME guidelines and will deposited in MIAME upon acceptance of the manuscript.

### Bioinformatics and data analysis

Gene ontologies were investigated with the g:profiler web based program (http:// biit.cs.ut.ee/gprofiler/) [25], using the GO database with status from October 18th 2011.

The microarray data analysis workflow was assembled using the Konstanz Information Miner open source software (KNIME; www.knime.org [26]). The raw data was preprocessed using Robust Multiarray Analysis (RMA) [27]. Background correction, quantile normalization, and summarization were applied to all expression data samples, using the RMA function from the *affy* package of Bioconductor [28], [29]. Low-expression genes with a signal below an intensity of 64 in any one of the 12 conditions were filtered out. The *limma* package (R & Bioconductor) was used to identify differentially expressed genes [27], with hESC set as control group. The moderated *t*-statistics was used for assessing the raw significance of differentially expressed genes (NCP versus hESC, hESC versus NEP). Then, final p-values were derived by using the Benjamini-Hochberg method to control the false discovery rate (FDR) [30] due to multiple hypothesis testing. Transcripts with FDR adjusted p-value of ≤0.05 and a fold change values ≥|2| were considered significantly regulated. The hierarchical clustering analysis was performed as previously described [31]. Average linkage was used as agglomeration rule for the clustering analysis. Distances based on the Pearson's correlation coefficient was used to group together transcripts with similar expression patterns across samples (rows of the heat map). Distances based on Spearman's rank correlations of the gene expression values was used to measure the similarity between samples. Then expression values within each row were normalized as Z-factors, and color-coded accordingly.

For the visualization of qPCR data, generated with the ΔΔCt method, we implemented a heat map solution as graphical representation. To express gene regulation, we used 256 steps for blue (down-regulation) and red (up-regulation). The scaling was adapted so that a manually chosen threshold value in each group (e.g. 20-fold up-regulation) defined the maximum color saturation. Then, color scaling steps were linearly mapped to gene regulation values between 1 and the threshold value in red and below 1 in blue. Genes regulated not significantly after FDR-correction were set to 1, were colored white and marked with letter “N” for not regulated.

## Results

### Chromatin changes during the differentiation of hESC to neuroectodermal progenitor cells

In order to investigate chromatin alterations and genetic regulation during initial neural differentiation we used the recently described hESC differentiation protocol towards neural epithelial progenitor cells (NEP) [17]. Using this differentiation procedure we obtained a pure and homogeneous cell populations in a fast and synchronized manner.

**Figure 1:**
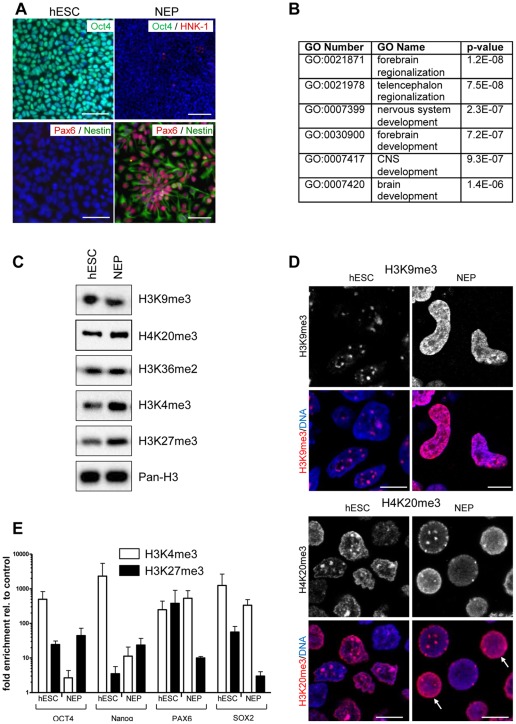
Effect of neuroectodermal differentiation on localization of histone marks. (A) hESC were differentiated towards NEP and stained with antibodies specific for Oct4, HNK-1 (neural crest marker), Pax6 (NEP marker) and nestin (neural stem cell marker). Nuclei were stained with the DNA dye H-33342 (blue). Scale bars: 100 µm. (B) GO analysis of the up-regulated genes in NEP compared to hESC (C) Whole cell extracts from hESC and NEP were analyzed by Western blot with antibodies specific for the indicated histone H3 modifications. Total histone H3 (Pan-H3) was used as loading control. (D) hESC and differentiated NEP were grown on glass cover slips and immunostained with antibodies specific for H3K9me3 or H4K20me3. The upper panels show grey-scale signal intensities of the stain, the lower panels show a superimposition of the same histone stain as above (red) with a DNA counter-stain (DAPI, blue). Arrows mark two cells with a diffuse H4K20me3 stain, which differs from the spot-like pattern always observed in hESC. Scale bars: 10 µm. (E) Chromatin immunoprecipitiation was perfomed from nuclei of hESC or NEP with antibodies specific for H3K4me3 and H3K27me3. The abundance of promoter regions of *OCT4, NANOG, PAX6* and *SOX2* was measured by qPCR with specific primers for the indicated genes. Data were compared to control samples prepared without specific antibody and are indicated as relative enrichment. Data are means ± SD from 2 experiments.

Immunostaining was used for the characterization of culture homogeneity. The hESC marker OCT4 was expressed in undifferentiated hESC, but was not detectable in NEP (Fig. 1A). Staining for the neural stem cell marker nestin, and for the NEP marker PAX6 was observed in >90% of all NEP, but not in hESC. As described by Chambers and colleagues [17], our differentiated NEP culture contained <2% of cells positive for the neural crest marker HNK1 (Fig. 1A). qPCR analysis of several neurodevelopmental markers also indicated that a neuroectodermal cell population had been obtained from hESC. For instance, *PAX6* and the neural regulator gene *NeuroD1* [32] were up-regulated in NEP >500-fold compared to hESC (Fig. S2, S3). Altogether, the phenotypic control of the differentiation procedure indicates that we had obtained a relatively pure population of NEPs.

To further validate this differentiation on gene expression level, we obtained a genome-wide expression profile by microarray analysis. The statistically most over-represented gene ontologies (GO), amongst the genes that were up-regulated in NEP more than 10 fold were all related to nervous system development (Fig. 1B). The six most significant GOs yielded for instance the categories “forebrain regionalization and development” and ”nervous system development”. This is in good agreement with previous data describing this NEP population as rostral committed CNS progenitor cells [17].

Transcriptional changes during the differentiation process are expected to be associated with epigenetic changes as it is known that epigenetic processes play a crucial role during cell differentiation [33]. In order to characterize such changes in our particular model system, we used various methods to characterize histone modifications on different levels of cell organization. First, we used Western blot analysis to quantify the global amount of different histone modifications across the entire chromatin (Fig. 1C). The overall levels of the various histone lysine modifications evaluated for this purpose remained constant during the differentiation independent of their property to activate (H3K36me2, H3K4me3) or silence (H3K9me3, H3K27me3, H4K20me3) gene expression. Secondly, the distribution of the heterochromatin marker H3K9me3 within the nucleus was analyzed by immunocytochemistry. The results showed, that a dramatic redistribution within the nucleus occurred. In hESC, H3K9me3 was localized in sharply-demarcated speckles, while the staining was diffuse in NEP (Fig. 1D). This relocalization process was confirmed by staining for another heterochromatin marker, H4K20me3 (Fig. 1D). In contrast, H3K27me3 and euchromatin marks (H3K4me3, H3K36me3, H3K9Ac) did not change their localization pattern upon differentiation (data not shown). In contrast to the western blot results the H3K9me3 immunostaining looks as if H3K9me3 would increase upon differentiation. This apparent discrepancy could either be due to the very dense chromatin structure in the H3K9me3 spots in hESC or due to high concentrations of H3K9me3 present in these heterochromatin spots that are not adequately reflected by the immunostaining method. Western blot analysis is in this case the more reliable method to quantify protein amounts, and therefore we conclude that there is no significant change in the overall extent of H3K9 methylation during differentiation. Thus, the altered spatial organization of the histone marks was not due to overall changes in the amount of heterochromatin and euchromatin. In a third approach, we looked for histone alterations on the level of individual genes. Chromatin immunoprecipitation was performed for histone marks typically associated with silenced (H3K27me3) or open gene promoters (H3K4me3). We have chosen these two histone marks, as developmental regulator genes are described to be in a poised state (ready for activation or silencing) through the simultaneous presence of H3K4me3 and H3K27me3 [34], [35]. The data indicated that the pluripotency genes *OCT4* and *NANOG* lost their H3K4 tri-methylation upon differentiation. Moreover, H3K27me3 was enriched in the *OCT4* promoter, whereas *NANOG* gained a bivalent status in NEP (Fig. 1E). Both types of changes are consistent with the silencing of their respective genes during the differentiation. In contrast, *PAX6* was bivalently modified in hESC, but lost the silencing H3K27me3 mark upon differentiation. This is in agreement with up-regulation of this gene in NEP. No such changes were found for the *SOX2* gene, which is active both in hESC and NEP (Fig. 1E). Altogether, these findings indicate, that chromatin may change locally (nuclear regional distribution) and gene-specifically, even though overall levels of certain chromatin marks (Fig. 1C) or of the DNA CpG methylation remain relatively constant [36]. We assumed that these specific epigenetic changes would require the fine-tuning of the activity of genes coding for enzymes that are specific for certain differentiation states or groups of genes. As little is known about such regulations, the major part of this study dealt with a characterization of epigenetic modifier transcripts, using neural differentiation as a test case.

### Compilation of a set of genes involved in chromatin modification

Initially, we used oligonucleotide high-density microarray-based whole transcriptome analysis in hESC and NEP for data mining concerning the regulation of epigenetic modifier genes. By manual screening for few candidates, we found for instance *DNMT3B* to be strongly down-regulated in NEP compared to hESC. This correlated well with the function of this gene as pluripotent stem cell marker [37]. The large and undefined amount of 400–600 (depending on definition or gene ontology included) known epigenetic regulator genes makes it difficult to search for them manually on a gene array data set. Also, systematic bioinformatic analysis did not reveal an overrepresentation of a GO related to chromatin modification. During this search process, we realized that a GO comprising all epigenetic modifiers has not yet been defined.

Therefore, we compiled and annotated here a set of such genes that is a representative cross-section of epigenetic modifiers. For an initial candidate list, we selected few positive controls such as neuronal specific BAF subunits (BAF53B, BAF60C) or PRMT8. These are either known to be expressed specifically in the brain or to have a proven function during neural development, at least in mouse model systems [16], [38]. Then, we scanned the GOs dealing with chromatin structure, histone modification, chromatin remodeling and DNA methylation. In combination with an extensive literature search we chose a representative set of these genes. Few additional factors were added due to their high sequence and domain homology to known epigenetic factors of the same family.

**Figure 2:**
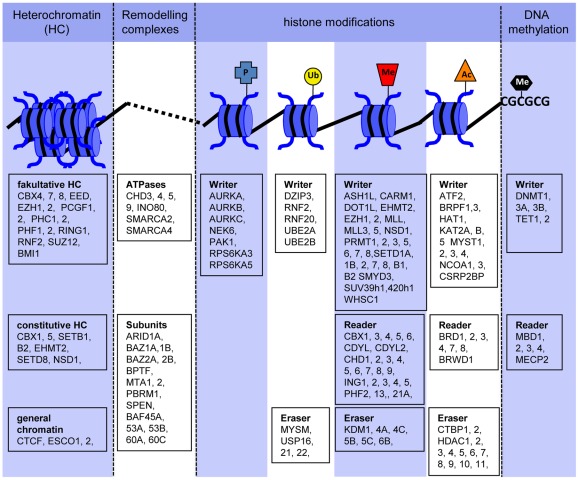
Compilation of 150 representative genes involved in epigenetic regulation. The genes were identified, selected, and classified according to their function based on published literature and database searches. In a further step, the genes related to histone modifications were sub-classified as writers (coding for modifying enzymes such as methyltransferases), as erasers (coding for de-modifying enzymes such as histone deacetylases) and as readers (coding for proteins that bind to the respective modification).

A total of 150 genes were selected according to the principles described above. These also comprised potential positive control markers for non-neuronal tissue, such as CDYL2 for the spleen [39], CHD9 for osteogenesis [40] or HDAC11 for oligodentrocyte differentiation [41]. The genes were classified according to their functional role in epigenetic processes, and a graphical overview of their mechanistic role was provided (Fig. 2). To make the background information for the selected genes broadly available, we compiled an extensive table that lists their supposed function as well as the supporting evidence for this function, together with relevant literature citations, and, if available, the genes' potential role in neurodevelopment (Fig. S4).

### Pronounced changes of the epigenetic regulator transcript profile during human neuroepithelial (NEP) differentiation

**Figure 3:**
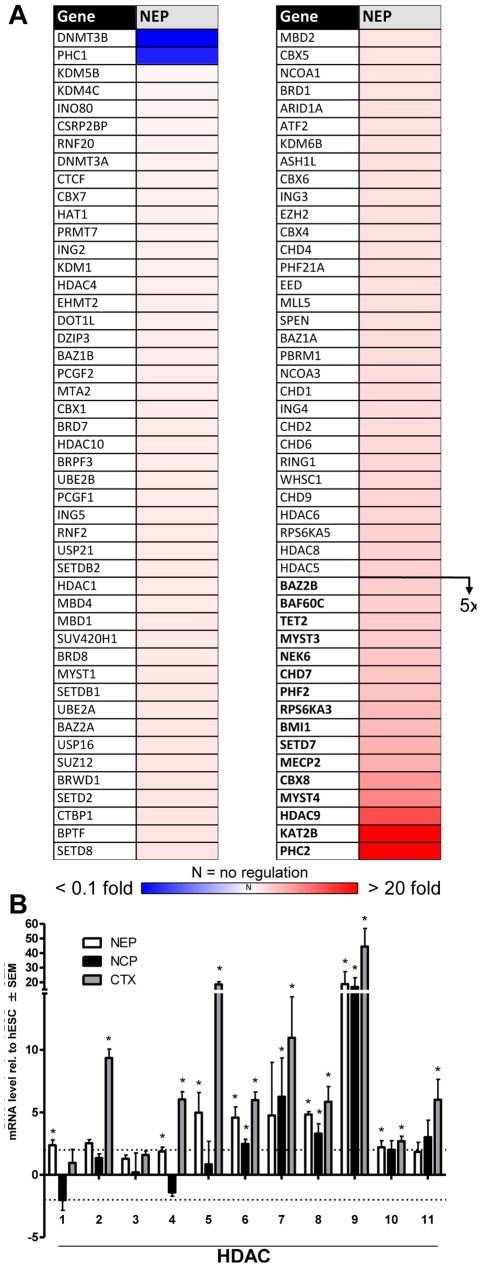
Transcriptional regulation of epigenetic modifiers during neuroepithelial differentiation. (A) The levels of epigenetic modifier transcripts of NEP and hESC were analyzed by qPCR in three independent cell preparations, and relative abundances were calculated. The data were color-coded, with up-regulated genes displayed in red and down-regulated genes in blue. Measures of variance and p-values are indicated in supplemental material, and only significantly regulated genes are displayed. Genes up-regulated >5-fold are displayed in bold. (B) The transcript levels of HDACs were determined for hESC, NEP, NCP and CTX. All expression levels of differentiated cells were normalized to those of hESC, and relative abundances are displayed. For instance, seven different HDACs were up-regulated in NEP compared to hESC. The dotted lines indicate 2-fold regulation levels. Data are means ± SEM of three independent differentiations. *: p<0.05

We searched manually for these 150 genes on our whole transcriptome microarray data set in hESC and NEP and found 22 genes to be regulated. This low amount of regulated genes was very surprising, in addition these genes were also regulated to a low level compared to neurodevelopmental genes (Fig. S3, S5). One reason for this could be due to the far lower sensitivity of microarray hybridization compared to qPCR. Therefore, we decided to use qPCR analysis as alternative approach. The relative expression changes of NEP vs. hESC were calculated from averaged data of three independent differentiations, and then visualized as a heat map (Fig. 3A). The full set of data including statistics is added as supplementary information (Fig. S5). About two thirds of the genes were up-regulated, and 16 of them reached relative transcript levels of 5-fold to 30-fold compared to hESC. For 68 of the genes, the relative expression levels identified by microarray and qPCR correlated well as 14 genes were up-regulated and 54 genes were not regulated with both methods. However, the regulation of an equally large group of genes (n = 72) was identified only by qPCR (Fig. S6). We conclude that qPCR is a more sensitive and specific approach for the quantification of expression levels of this hand-picked selection of epigenetic modifier genes. This was also supported by the observation that the absolute expression levels detected by qPCR were mainly below ten-fold which might not have been detected by microarray hybridization. The remaining 10 genes that showed contradictory expression levels in qPCR analysis compared to microarray hybridization might be outliers that were not removed by statistical analysis of the microarray data. Alternatively, this could also be due to wrong affymetrix annotations of these genes or due to the analysis of different transcripts (splicing) of the same gene. As one example for a class of enzymes regulated on the transcriptional level, we chose histone deacetylases. Eight out of the eleven isoforms present in our list were found to be up-regulated at least 2-fold by qPCR. *HDAC9* was regulated more than 20-fold (Fig. 3B, S6).

### Differentiation of hESC to neural crest precursor cells (NCP)

**Figure 4:**
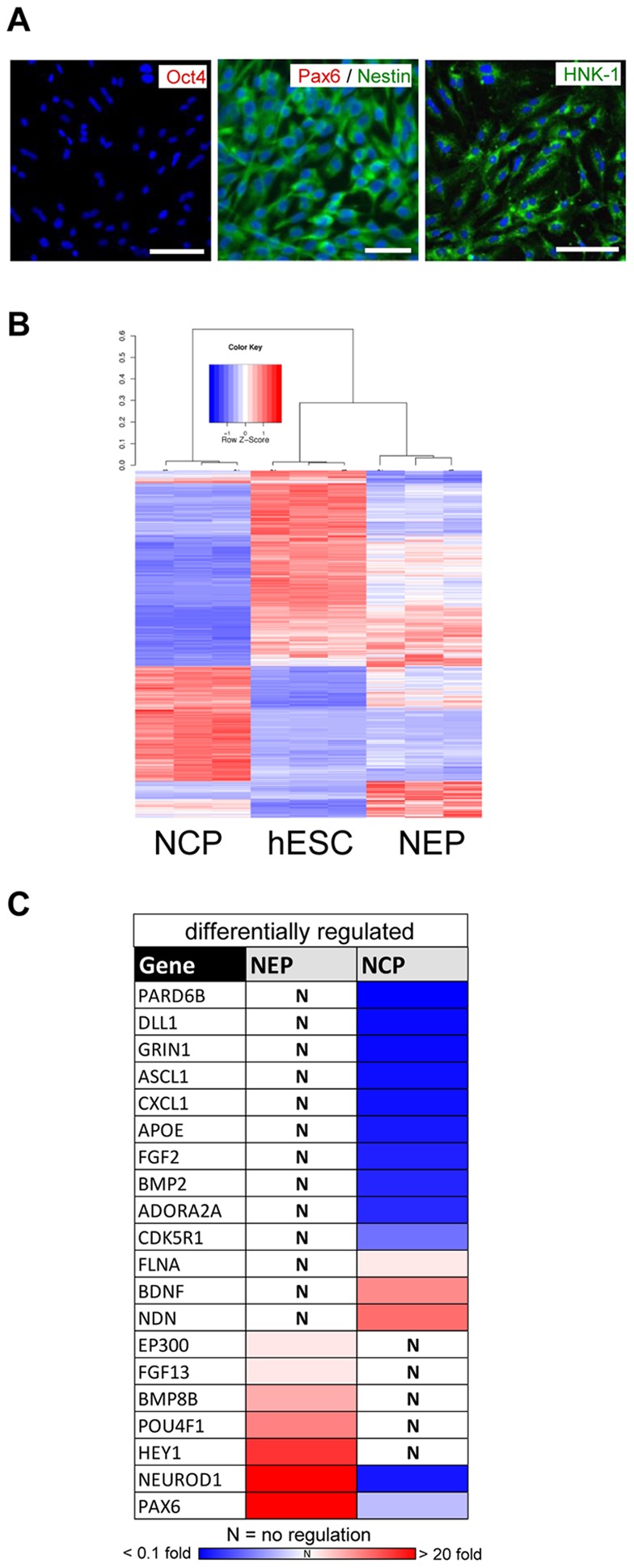
Alternative early neural differentiation to neural crest progenitors. (A) hESC were differentiated towards NCP and stained with antibodies specific for *OCT4* (no stain observed), *PAX6* (no stain observed), *NESTIN* and *HNK-1*. Cell nuclei were labeled with the DNA dye Hoechst H-33342 (blue). Scale bars: 100 µm. (B) Pairwise comparisons of hESC, NEP or NCP yielded 4277 differentially expressed transcripts. The heat map displays the genes after clustering according to the Pearson's correlation of their expression values across samples. The colors represent Z-scores of the row-wise normalized expression values for each gene. The dendrogram indicates the pattern similarities indicated by Spearman correlation distances (1- Spearman correlation coefficient) and shows a large separation of NCP from NEP and hESC. (C) The expression of early neuronal marker genes was measured in three preparations each of hESC, NEP and NCP by qPCR. The transcript levels of NEP and NCP were calculated relative to hESC. The relative gene expression levels were color coded (significant down-regulation vs. hESC in blue; significant up-regulation in red; non-significant changes marked by “N”. The genes showing different behavior in NEP vs NCP are displayed. All measures of variance and p-values are indicated in the supplemental material.

To obtain some information on the specificity of the changes observed, we generated a different neural precursor population. A recently described protocol was used to generate a homogeneous population of NCP [18], [19], which was characterized by immunocytochemistry. These peripheral nervous system precursors were negative for the pluripotency marker OCT4 and the NEP marker PAX6 (Fig. 4A). Instead, NCP were homogeneously positive for the neural crest marker HNK-1 and the general neural stem cell marker Nestin (Fig. 4A). Comparison of the whole-genome transcript pattern of NCP with that of hESC and NEP showed that we had generated a clearly distinct cell population. Clustering analysis indicated a strong separation of NCP from either hESC or NEP (Fig. 4B). The most-significantly overrepresented GOs of NCP comprised “nervous system development”, “extracellular matrix”, and “skeletal system development”. This correlates well with the role of NCP as precursor for several peripheral cell types, including cranial bone and cartilage [42]. In addition the hierarchical clustering revealed a high reproducibility between different hESC, NEP and NCP preparations.

To further confirm that we are differentiating hESC into two different cell types we performed a gene expression analysis by qPCR on 84 genes known to be involved in neurodevelopment (Fig. S2). A comparison of the differentially regulated genes (Fig. 4C) showed some pronounced differences between the cell types. Some genes (e.g. *PAX6*) were up-regulated in NEP, but not in NCP. Conversely, genes like *BDNF* were up-regulated in NCP, and down-regulated in NEP. Amongst the genes jointly up-regulated in NEP and NCP those that play a role in axonal growth and guidance (*PTN, NRP1, EFNB1*) [43], [44] were much stronger up-regulated in NCP than in NEP (Fig. S5). In summary these data show that NEP and NCP are indeed two different neural cell populations, although both are derived from the same cell source.

### Changes of the epigenetic regulator transcript profile during differentiation of human pluripotent stem cells to neural crest

**Figure 5:**
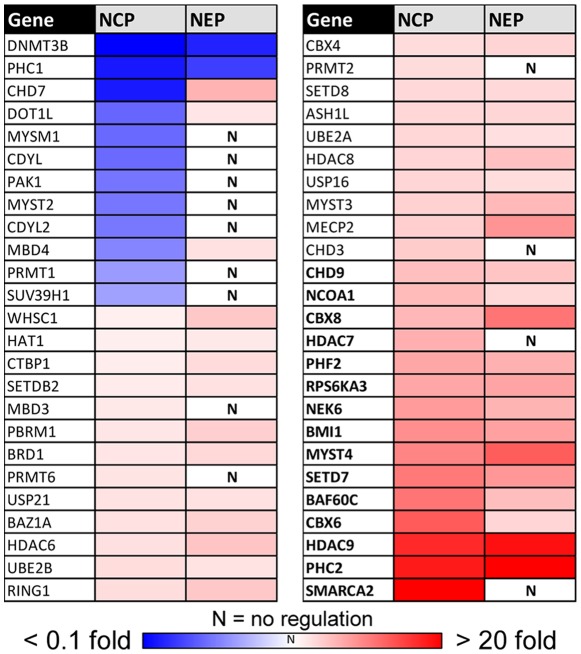
Regulation of epigenetic modifiers in NCP, and their comparison to NEP. The levels of epigenetic regulator gene transcripts were measured in hESC, NCP and NEP by qPCR, and the expression levels were calculated relative to the levels in hESC. All genes that showed significant up- or downregulation in NCP compared to hESC are displayed. The relative expression level (vs. hESC) was color coded, as illustrated in the chromatic scale in the bottom. Genes with >5-fold expression in NCP vs. hESC are shown in bold. For better comparison, the data for the same genes are shown for NEP in the right-hand column. Measures of variance and p-values are indicated in the supplemental material.

When the expression levels of the 150 epigenetic modifiers defined in figure 2 were measured in NCP, we found about 50 regulated genes and 15 transcripts with a relative abundance of >5-fold relative to hESC. Indeed, we found besides genes that are equally expressed also different genes expressed in the different cell types. 13 genes were identified to be significantly regulated in NCP, but not in NEP (Fig. 5), and 8 genes were up-regulated only in NEP (Fig. S5). Amongst the NCP-specific genes was *SMARCA2* (also called *Brm*), one of the ATPase subunits of the BAF complex [4]. The differential up-regulation of this factor was also evident from the microarray data (Fig. 4B; not shown), and this epigenetic modifier is known to play a role in neurodevelopment and its disorders [45]. A further differential regulation of chromatin remodeling ATPases was observed for *CHD7*, which was down-regulated in NCP, while it was up-regulated in NEP.

### Relative abundance of epigenetic regulator transcript levels in cortical neurons relative to stem cells and neural precursors

**Figure 6:**
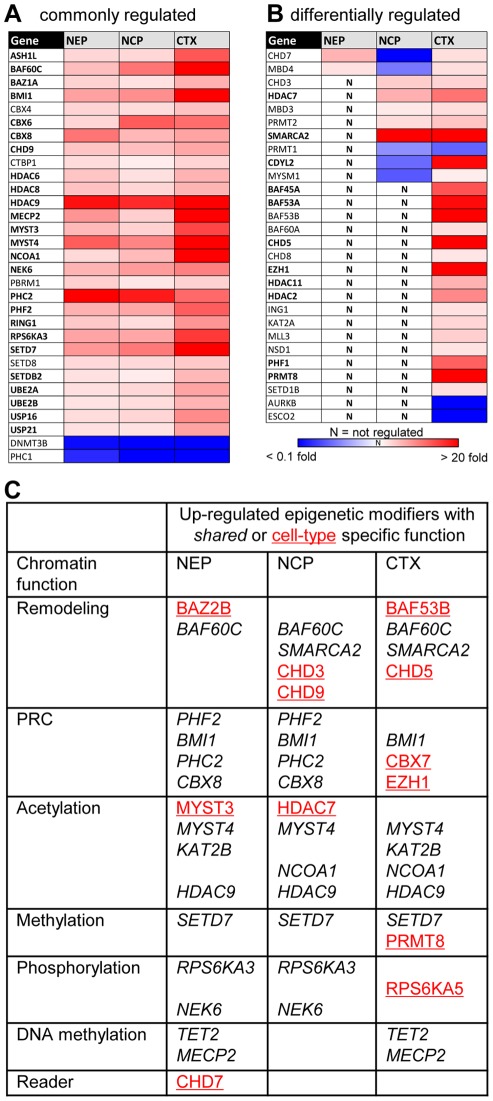
Comparison of epigenetic modifiers in cortical neurons with early neural precursors. The levels of epigenetic regulator gene transcripts were measured as in Fig. 5, but samples from human cortex (CTX) were included. Up-regulated genes are displayed in red, down-regulated transcripts in blue, as indicated by the chromatic scale. Genes with CTX transcript levels 5 times higher than in hESC are indicated in bold letters (A) Display of all genes regulated into the same direction in early neural precursors and mature neurons. (B) Differentially regulated genes. Measures of variance and p-values are indicated in the supplemental material. (C) Summary of the most up-regulated common (italics) or cell type-specific (red, underlined) chromatin modifier genes, sorted according to their function.

We complemented the studies on early neurodevelopment with a comparison of these cell populations to human cortical samples (Ctx). For normalization of the results and for comparability with the other cell populations, transcript levels in Ctx were displayed relative to those in hESC. Of the 150 studied genes, 54 were significantly higher expressed in Ctx, 5 showed lower expression than in hESC (Fig. 6, S5).

About 20% of all genes examined were expressed higher in all neural cell types (NEP, NCP, Ctx) than in hESC (Fig. 6A). Further 10% of the transcripts studied (n = 16) were only up-regulated in Ctx. These comprised for instance the arginine methyltransferase PRMT8 [38] and BAF53B [16], *bona fide* examples of brain-specific chromatin modifiers in other species (Fig. 6B). For a better overview we displayed conspicuous cell type-specific genes, and examples of those shared by more than one cell type in a table, in which we sorted the genes according to their functional role in epigenetic regulation (Fig. 6C). Interestingly, only few of these genes are involved in histone methylation, phosphorylation or DNA methylation. Although histone lysine methylation represents the largest group of genes in our set of epigenetic modifiers, we found only SETD7, a H3K4 HMT (histone methyl transferase), to be differentially expressed in neural cells compared to hESC.

**Figure 7:**
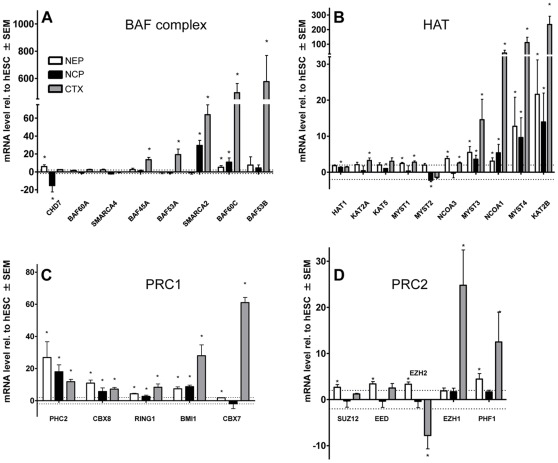
Synopsis of the regulation of different epigenetic modifier groups at different stages of neuronal differentiation. Four groups of epigenetic modifiers were selected for a comparison of relative expression levels of NEP, NCP and CTX. Data were obtained, and significances calculated as described earlier. All data are means ± SEM of three independent differentiations. (A) Genes that code for subunits of the BAF remodeling complex. (B) Genes that code for histone acetyl transferases (HAT). (C, D) Genes that are involved in PRC1 and PRC2 complex formation. *: p<0.05 vs hESC transcript level.

The three main groups of differentially expressed genes related to chromatin remodeling, polycomb complexes and histone acetylation. Therefore we assembled all investigated genes involved in these three epigenetic processes. First, the expression levels of components of BAF remodeling complexes were compared (Fig. 7A). Interestingly, the subunits considered to be specific for neural stem cells (BAF45A, BAF53A) were found to be highly expressed in cortex samples. However, the neuron-specific subunits (BAF60C and BAF53B) were expressed in Ctx at more than an order of magnitude higher levels (several hundred-fold compared to hESC) than BAF45A/BAF53A. A low, but significant expression of BAF60C was here also observed in NEP and NCP. This is in agreement with the literature as expression of this subunit has also been described for murine neural progenitor cells [16].

Next, we compared the expression levels of histone acetyl transferases (HAT) and found pronounced up-regulations of KAT2B (PCAF), MYST3 (MOZ), MYST4 (MORF) and NCOA1 (compared to hESC) in the other three cell types (Fig. 7B). This may indicate a role in general neuro-development. Such a role has indeed been shown for MYST4 and NCOA1 in murine model systems. MYST4 was shown to be essential for neuronal development of mouse cortex [46] and NCOA1 is elevated in murine neural stem cells [47].

One of the most important epigenetic developmental regulators is represented by the polycomb group family of proteins. These proteins assemble two main complexes: PRC2 that methylates H3K27 via the catalytic subunit EZH2 or EZH1, and PRC1 that binds to H3K27me via the CBX4 (Pc2) subunit, ubiquitinates H2AK119 and establishes the dense heterochromatin structure that is needed for gene silencing [48]. Among the components of PRC1, we found a particular regulation pattern that is potentially specific for the developmental stage and/or tissue: CBX7, a homologue to CBX4 (Pc2) was up-regulated only in Ctx, but not in NEP or NCP. In addition, CBX8, a further particular isoform of CBX4, was up-regulated in all three cell types (Fig. 7C). This may indicate a neuron-specific role for CBX7 and a more general neuronal role for CBX8.

Most interestingly, we found a pronounced differential regulation pattern for the two isoforms of the PRC2-associated H3K27 methyl transferase. In Ctx, EZH2 was down-regulated, while EZH1 was strongly up-regulated (Fig. 7D). For murine *in vitro* and *in vivo* systems it was reported that EZH2 is exchanged with EZH1 in fully differentiated non-proliferative tissue [49]. Moreover, the methylation of H3K27 catalyzed by EZH1-containing PRC2 complex results in a very dense chromatin structure, while chromatin methylated at H3K27 by EZH2-PRC2 does not change its higher order structure significantly [49].

## Discussion

In this study, we used two homogeneous and well-controlled neural differentiation systems to examine hitherto unknown regulations of the large set of genes coding for epigenetic regulators. The early precursor cells were further compared to cortical tissue. Thus, the cell types used to explore changes of chromatin modulators on the transcriptional level spanned developmental stages from embryonic stem cells to post-mitotic neurons. Our findings of pronounced cell type-specific regulations of the transcripts coding for chromatin-modifying proteins complement other types of approaches to study epigenetic regulations. While most previous studies have focused on the chromatin itself, by investigating histone modifications, DNA methylation patterns or the spatial chromatin structure, we investigated the expression levels of the genes catalyzing the above mentioned chromatin alterations.

For the initial characterization of the experimental systems used, we chose several traditional endpoints. For instance, some histone modifications were studied directly by Western blot and immunostaining. Although the global amount of methylated histones did not change during neural differentiation, the sub-nuclear distribution of such histone marks, as well as their abundance on individual promoters underwent striking changes. In parallel with the down-regulation of the stem cell markers *OCT4* and *NANOG* upon differentiation of hESC to NEP, the enrichment of H3K4me3 in their promoter region was reduced. In the same vein, the up-regulation of the neuroectoderm marker *PAX6* was paralleled by a loss of the repressing H3K27me3 mark in its promoter (Fig. 1). However, very little is known about the transcriptional regulation of the enzymes responsible for such chromatin changes. Because the group of genes involved in epigenetic modification has been poorly defined, only few broad tissue specific expression studies have been performed [9], [10], [11], and these are mostly limited to mouse models or lower vertebrates. The set of epigenetic modifiers selected and characterized here should help to facilitate more studies on human tissue expression.

Many different proteins have been described to be directly or indirectly involved in opening or closing the chromatin structure, and therefore providing or inhibiting access for the transcriptional machinery. Complexity is added by synergistic and antagonistic actions of many factors, by the formation of multi-protein complexes with exchangeable subunits, and by the involvement of one given factor in multiple complexes and reactions [4], [16]. Particularly important for our study is the feature that often one specific chromatin modification can be set by several different enzymes. For example, more than 15 different histone methyltransferases are known to catalyze the methylation of lysine 4 of H3 [3]. Therefore it was interesting, that among the 8 H3K4 HMT, only the H3K4 histone methyltransferase *SETD7* was strongly up-regulated in both the neural precursor populations and in cortical tissue (Fig. 6). This indicates that this specific enzyme may play a particular role in regulating neural genes.

Also the BAF chromatin remodeler multi-protein complexes contain different alternative ATPase subunits, such as SMARCA2 and SMARCA4 [4]. It is still unclear, under which circumstances these subunits are assembled into the BAF complex. Here we found cell-type specific regulation of these subunits (Fig. 6). The differential use and transcriptional regulation of chromatin remodelers may help in the future to characterize different cell types and developmental stages. Combined with more detailed future knowledge of preferred targets, e.g. of SETD7 or SMARCA2-containing complexes, cell-specific maps of their transcriptional regulation may complement other approaches to yield information on differential epigenetic regulations. The foremost outcome of the suggested PCR-based analysis will be a fast and efficient strategy for screening different tissues or cell types for differences of regulation on the transcriptional level. This may yield interesting fingerprints as such. In addition, this approach could be used as a first screen to identify candidates for more in depth examination of epigenetic modifiers. The subsequent studies would then address the functional relevance of the gene products. This would require more information on protein levels, on post-translational modifications of the respective proteins and also of their association with different multi-protein complexes.

During the course of this study, we tested different approaches to define a useful set of epigenetic modifiers. Initially, we looked up the genes that are included in the most obvious epigenetic GOs (“histone modification”, “chromatin organization”, “chromatin remodeling”). We realized that many of the genes included in these GOs did not fulfill our criteria for an epigenetic modifier, i.e. the literature did not support their direct role in epigenetic modifications or regulations. For example, the genes coding for nitric oxide synthase or IL-1 were included in this list. Study of the primary literature for many more genes showed that the supporting evidence for a predominant role in epigenetics was often not solid. Part of the information included in the GO database is assembled in initial stages by data mining algorithms that screen scientific publications according to key words. This approach works very well for clearly defined biological areas such as “carbohydrate metabolism”. It works less well for epigenetic regulator genes, as many different processes impact on epigenetics and *vice versa*. This can easily lead to an erroneous classification of an epigenetically regulated gene as epigenetic regulator. Moreover, many enzymes like the above-mentioned nitric oxide synthase may indeed affect chromatin modifiers indirectly [50], although this may not be their major physiological role.

Due to these problems, we hand-picked here a set of 150 epigenetic regulators genes after careful study of the literature. The list consists of players involved in all major groups of epigenetic mechanisms. We are aware of the fact that a more exhaustive list would rather contain about 1000 genes. Such a list would certainly be more complete, but it would also contain several disputable candidates, and it would be much harder to handle. We took here the practical approach to validate the applicability of our set of genes to confirm some known candidates. Our list included some epigenetic regulator genes that have known roles in vertebrate and non-vertebrate neuronal development or are expressed specifically in the brain in mouse model systems. The relative expression levels of these genes in our study provided first evidence for the usefulness of our set of genes. For instance, our data showed that *PRMT8*, *BAF53B* and *CHD5* were exclusively or very highly expressed in human cortex tissue (out of the cell types examined). This agrees with information for mouse tissue [16], [38], [51], and we show here for the first time that these genes are also highly expressed in human brain.

At present, very little is known about epigenetic regulator genes that may be specific for non-neuronal tissues. Most knowledge is derived from knockout mice that show developmental defects upon knock down of a specific regulator gene. On this basis, some candidates in our list of genes may be up-regulated in other differentiation systems, during the generating of other cell types/tissues. For instance, CHD9 was reported to be involved in osteogenic cell differentiation [40], and CDYL2 is expressed highly in testis, prostate, spleen and leukocytes [39]. For the examination of new tissues, these may be used as positive controls. However, this will have to be paralleled by the choice of a set of tissue-specific differentiation markers (similar to *PAX6*, *Nestin* and *NeuroD1* for NEP), which can then be used as overall positive controls for the correct differentiation and correct analysis procedure.

We also tested the ability of the gene modifier set to identify new differential patterns in related cell populations, such as different neural precursors or neural precursors vs. post-mitotic neurons. As we identified, in general, astonishing cell type differences, and differential expression patterns, for the genes that are yet little characterized, we conclude that the chosen set of modifiers represents a useful tool and starting point for future studies.

The major groups of genes that we found to be highly-expressed throughout neuronal development, were involved in histone acetylation, chromatin remodelling and PRC complex components. Surprisingly, only one H3K4 histone methyl transferase, *SETD7*, was strongly regulated. Further interesting changes involved ATPase subunits of chromatin remodelling complexes: *CHD7* and *SMARCA2* showed cell-specific expression profiles. In NEP, *CHD7* was up- and in NCP it was down-regulated. It is known that this chromodomain protein is required for the differentiation of hESC to early NCP and for frog neural crest formation [52]. However, it is unclear, whether the gene still plays a role in developed neural crest cells or their progeny. Our findings of a relative down-regulation (also confirmed by microarray analysis; not shown) may indicate that CHD7 is less important, once NCP have developed, expanded and undergone epithelial-to-mesenchymal transition (as the cells used here). In contrast to *CHD7*, *SMARCA2* was up-regulated in NCP but not in NEP. This data may indicate a role of the ATPase in NCP cells, but we are aware of the fact that knowledge on protein levels and functional data would be required for a more definite statement.

Another example for cell type-specific expression differences was found for members of the PRC1 complex. The canonical *CBX4* (Pc2) subunit of PRC1, that binds the H3K27me mark [48], [53], was neither regulated during early differentiations towards NEP and NCP nor was it highly expressed in the cortex. Instead, we found *CBX8* to be up-regulated in all investigated cell types, and *CBX7* to be highly expressed in cortex, while the levels in other cells resembled those in hESC. Therefore, the CBX4 homologue CBX7, may have a specific role in adult brain. We also obtained evidence suggesting a cell-type-specific subunit switch in the PRC2 complex. *EZH2*, the methyl transferase catalyzing the H3K27me3 modification, is expressed in the two progenitor cell types (NEP and NCP), but its expression was low in cortex, compared to hESC. Instead, its isoform *EZH1* was highly expressed in the adult tissue. Interestingly, it has been shown for mouse cells that chromatin methylated by EZH1 had a much denser structure than chromatin methylated by EZH2 [49]. However, the exact function of EZH1 is not fully clarified yet. Recently, EZH1 was reported to co-localize with active histone modification H3K4me3 indicating a different function of PRC2-EZH1 complex [54].

In summary, we compiled a representative set of 150 genes involved in epigenetic regulation of gene expression. This list is only a small reflection of the hundreds or even up to thousand genes that have been described to be involved in epigenetics. The set presented here can still be handled easily, and it has been shown to be useful to indicate changes due to differentiation. This provides a basis for more detailed investigations of certain candidates or more comprehensive investigation of certain groups of genes. With this study, we have added a new approach to characterize the epigenetic status of a cell or tissue. It may be comparable to the characterization of transcription factor networks [55], [56] or transcriptional profiling in other areas of cell biology [24], [57], [58], [59]. In all such cases the information on the actual state of cellular constituents (in our case: chromatin) is very limited, and the absolute expression levels of what is measured are hard to interpret, but the relative changes and their dynamics can yield useful descriptions of a biological system, and define a level of information not easily obtained by other methods. This may be particularly interesting for toxicology, for the characterization of diseased tissue, and for the comparison of different cell populations generated *in vitro*.

## Supporting Information

Figure S1 **Antibodies used for immunstaining and primers used for qPCR.**
(PDF)

Figure S2 **Neurodevelopmental genes examined in this study.** The neurodevelopmental genes investigated in this study by qPCR are listed alphabetically by their gene symbol. The cDNAs are defined by the respective RefSeq accession numbers (as used in NCBI data bases). Alternative gene names frequently used in the literature have been added in paretheses. The references provide further background information.
(PDF)

Figure S3 **Expression of neuro-developmental genes in NEP and NCP.** hESC were differentiated into NEP or NCP. RNA was prepared from all types and qPCR was performed using primers specific for the indicated neurodevelopmental regulator genes. Threshold cycle values (Ct) were measured with a Biorad light cycler. Ct values were normalized to house keeping genes, and relative gene expressions were calculated by normalization to hESC expression levels. Data are means of three independent differentiations +/− standart deviation (SD). p-values were calculated with Studens t-test and corrected for false discovery rate (FDR) according to Benjamini-Hochberg. They correspond to the statistical difference from the expression levels in hESC. Data corresponds to Fig. 4C.
(PDF)

Figure S4 **150 Epigenetic regulators examined in this study.** The epigenetic regulator genes investigated in this study by qPCR are listed alphabetically by their gene symbol. The cDNAs are defined by the respective RefSeq accession numbers (as used in NCBI data bases). Alternative gene names frequently used in the literature have been added in paretheses. The references provide further background information.
(PDF)

Figure S5 **Expression of epigenetic regulators normalized to hESC.** hESC were differentiated into NEP or NCP. RNA was prepared from all types or cortical tissue samples. qPCR was performed using primers specific for the indicated neurodevelopmental regulator genes. Threshold cycle values (Ct) were measured with a Biorad light cycler. Ct values were normalized to house keeping genes, and relative gene expressions were calculated by normalization to hESC expression levels. Data are means of three independent differentiations +/− standart deviation (SD). p-values were calculated with Studens t-test and corrected for false discovery rate (FDR) according to Benjamini-Hochberg. They correspond to the statistical difference from the expression levels in hESC. Data corresponds to Fig. 2, 5, 6, and 7.
(PDF)

Figure S6 **Comparison of relative expression data from microarray and qPCR.** hESC were differentiated into NEP, and RNA was prepared from undifferentiated hESC and NEP. qPCR was performed using primers specific for the 150 epigenetic regulator genes and threshold cycle values (Ct) were measured with a Biorad light cycler. Ct values of NEP were first normalized to house keeping genes. Fold expression levels were obtained by further normalization to hESC (Fig. S5). RNA for microarray was prepared as described above and hybridization on Affymetrix gene chips was perfomed. After bioinformatic analysis, we obtained expression levels relative to hESC. Microarray data were screened for our set of 150 epigenetic regulator genes investigated by qPCR. Fold expression values obtained from microarray data are blotted on the y-axis, and fold expression levels obtained from qPCR are blotted on the x-axis. For both data sets, non- significant values were set to 0. n: amount of genes in the different groups. Dotted lines indicate 2-fold regulations.
(PDF)
