# Supplementary material for: Extensive Transcriptional Regulation of Chromatin Modifiers during Human Neurodevelopment
Source: PLoS One. 2012 May 9;7(5):e36708. doi: 10.1371/journal.pone.0036708 (PMC3348879; doi:10.1371/journal.pone.0036708)
Supplement: Figure S1 — Antibodies used for immunstaining and primers used for qPCR. (PDF) [file pone.0036708.s001.pdf]

Figure S1: Antibodies used for immunstaining and primers used for qPCR

Antigen	Antibody (supplier)		Dilution	Species
Antibodies for immuno staining				
Oct4	anti-Oct4 (Santa Cruz)	sc-5279	1:200	mouse
Nestin	anti-nestin (R&D Systems)	MAB1259	1:200	mouse
CD57/HNK-1	anti-HNK-1 (Sigma)	C6680	1:200	mouse
Pax6	anti-Pax6 (Covance)	PRB-278P	1:200	rabbit
H3K9me3	anti-H3K9me3 (Millipore)	07-442	1:200	rabbit
H4K20me3	anti-H3K20me3 (Active Motif)	39181	1:200	mouse
Antibodies for western blot				
H3K9me3	anti-H3K9me3 (Millipore)	07-442	1:1000	rabbit
H3K36me2	anti-H3K36me2 (cell signalling)	9758	1:1000	rabbit
H4K20me3	anti-H3K20me3 (Active Motif)	39181	1:1000	mouse
H3K27me3	anti-H3K27me3 (Millipore)	07-449	1:1000	rabbit
H3K4me3	anti-H3K4me3 (Active Motif)	39915	1:1000	rabbit
Pan-H3	anti-H3 (Abcam)	ab1791	1:5000	rabbit
Antibodies for ChIP				
H3K4me3	anti-H3K4me3 (Millipore)	17-614		rabbit
H3K27me3	anti-H3K27me3 (Active Motif)	39535		rabbit

Primer	Sequence (5`→3`)	amplicon length (bp)
Primers for gene expression analysis		
BAF45A for	CGAGGTGATGAGAAACGGAAAAATAAAGG	194
BAF45A rev	GGCTTGTACCCAGGAACTGACTT	194
BAF53A for	CAGGTCTCTATGGCAGTGTAATAGTGGC	190
BAF53A rev	GTACCCAAAGAGGCTAGAATGGAG	190
BAF53B for	TGATCGCAGCCAAGGAG	175
BAF53B rev	GCAGCCACCTGTTCATCGTAGG	175
BAF60A for	CAGGGTTTCATCAATGACTGGCTTCAG	154
BAF60A rev	GCACCTTGGAGTAGAAGTATCGGCA	154
BAF60C for	GGTGGAGGAGCCATTAAAGGGG	189
BAF60C rev	GAGCGGAGCAGGTCTTGGAC	189
EZH1 for	GCTTCCTTCACCCTTTTCATGCCACCC	163
EZH1 rev	CGACGACCAGAGCACTTGGAG	163
TET1 for	TGCCAACCTTAGGGAGTAACACTG	172
TET1 rev	GCAGTCTTCGGGGACCAGGA	172
TET2 for	CCTATTGCTAAGTGGGTGGTTCGC	156
TET2 rev	GTAGAGTTTGTCAGCCAGAGACAGC	156
Primers for ChIP analysis		
Oct4 for	GAGGATGGCAAGCTGAGAAA	148
Oct4 rev	CTCAATCCCCAGGACAGAAC	148
Nanog for	GTTCTGTTGCTCGGTTTTCT	95
Nanog rev	TCCCGTCTACCAGTCTCACC	95
Pax6 for	AAGGGAACCGTGGCTCGG	96
Pax6 rev	ATTAGCGAAGCCTGACCTCTG	96
Sox2 for	GCACCCCACTGAAACTCTTC	130
Sox2 rev	TGCAGGATCTCAGTGTTTCG	130
