# Supplementary material for: Extensive Transcriptional Regulation of Chromatin Modifiers during Human Neurodevelopment
Source: PLoS One. 2012 May 9;7(5):e36708. doi: 10.1371/journal.pone.0036708 (PMC3348879; doi:10.1371/journal.pone.0036708)
Supplement: Figure S2 — Neurodevelopmental genes examined in this study. The neurodevelopmental genes investigated in this study by qPCR are listed alphabetically by their gene symbol. The cDNAs are defined by the respective RefSeq accession numbers (as used in NCBI data bases). Alternative gene names frequently used in the literature have been added in paretheses. The references provide further background information. (PDF) [file pone.0036708.s002.pdf]

Figure S2: Neurodevelopmental genes examined in this study

Gene	Description	Ref	Accession
ACHE	acetylcholinesterase	[1]	NM_000665
ADORA1	adenosine receptor A1, belongs to the G-protein coupled receptor 1 family	[2]	NM_000674
ADORA2A	adenosine receptor A2a, belongs to the G-protein coupled receptor 1 family	[2]	NM_000675
ALK	receptor tyrosine kinase expressed specifically in the nervous system	[3]	NM_004304
APBB1	multimodular adaptor protein, binds to APP; required for synaptogenesis	[4]	NM_001164
APOE	apolipoprotein E; associated with Alzheimer´s disease	[5]	NM_000041
ARNT2	neuronal transcription factor, regulating cell cycle progression and preventing cell death	[6]	NM_014862
ARTN	artemin; ligand for the RET receptor and uses GFR-alpha 3 as a coreceptor	[7]	NM_003976
ASCL1 (MASH1)	proneural transcription factor; dopaminergic differentiation	[8]	NM_004316
BAI1	P53-target gene specifically expressed in the brain; expressed in neuronal cells of the cortex but not in astrocytes	[9]	NM_001702
BDNF	brain-derived neurotrophic factor	[10]	NM_001709
BMP15	member of the bone morphogenetic protein family which is part of the transforming growth factor-beta superfamily	[11]	NM_005448
BMP2	member of the bone morphogenetic protein family which is part of the transforming growth factor-beta superfamily	[12]	NM_001200
BMP4	member of the bone morphogenetic protein family which is part of the transforming growth factor-beta superfamily	[12]	NM_130851
BMP8B	member of the bone morphogenetic protein family which is part of the transforming growth factor-beta superfamily	[13]	NM_001720
CDK5R1 (p35)	activator of CDK5, which is involved in neuronal migration during CNS development, synaptic activity in matured neurons, and neuronal cell death in neurodeg. diseases	[14]	NM_003885
CDK5RAP1 (C42)	binds CDK5R1	[15]	NM_016408
CDK5RAP2 (C48)	binds CDK5R1	[15]	NM_018249
CDK5RAP3 (C53)	binds CDK5R1	[15]	NM_176096
CHRM2	muscarinic cholinergic receptor	[16]	NM_000739
CXCL1	C-X-C-motif chemokine 1	[17]	NM_001511
DLG4 (PSD95)	linked to NMDA reseptors at synaptic sites, key role in mediating trafficking, clustering, and downstream signaling events, following receptor activation	[18]	NM_001365
DLL1	expression is found in mature DA neurons of the substantia nigra (SN) and ventral tegmental area (VTA); role in differentiation	([19]	NM_005618
DRD1	dopamine receptor D1; expressed postsynaptically on dopamine target cells	[20]	NM_000794

Gene	Description	Ref	Accession
DRD2	dopamine receptor D2; expressed postsynaptically on dopamine target cells and presynaptically on dopaminergic neurons	[20]	NM_000795
DVL3	axonal specification, neurite outgrowth	[21]	NM_004423
EFNB1	presynaptic development: Ephrin-B1 (EFNB1) and EFNB2 function with EphB to mediate presynaptic development via syntenin-1	[22]	NM_004429
EGF	epidermal growth factor; involved in growth, proliferation and differentiation	[23]	NM_001963
EP300	histone acetyltransferase p300; regulates transcription via chromatin remodeling, important in the processes of cell proliferation and differentiation	[24]	NM_001429
ERBB2	member of the epidermal growth factor (EGF) receptor family of receptor tyrosine kinases; roles in neuronal migration, axon guidance and synaptic plasticity	[25]	NM_004448
FEZ1	interaction of FEZ1 with microtubules was shown to promote neurite extension	[26]	NM_005103
FGF13	fibroblast growth factor 13	[27]	NM_004114
FGF2 (BFGF)	basic fibroblast growth factor	[28]	NM_002006
FLNA	neuronal migration	[29]	NM_001456
GDNF	ability to promote survival of mesencephalic DA neurons	[30]	NM_000514
GNAO1	guanine nucleotide-binding protein G(o) subunit alpha; involved in transmembrane signaling	[31]	NM_020988
GPI	glucose-6-phosphate isomerase	[32]	NM_000175
GRIN1 (NMDAR1)	one of the major mediators of excitatory neurotransmission in the central nervous system	[18]	NM_007327
HDAC4	histone deacetylation, class IIa HDAC, highly expressed in brain, can be neuroprotective	[33]	NM_006037
HDAC7	histone deacetylation, class IIa HDAC, class IIa HDACs interact with the DNA-binding domains of MEF2 proteins and convert them from activators to repressors	[34]	NM_016596
HES1	member of basic helix-loop-helix family of transcription factors; transcriptional repressor	[35]	NM_005524
HEY1	member of the hairy and enhancer of split-related (HESR) family of basic helix-loop-helix (bHLH)-type transcriptional factors, Notch target gene, not neuro-specfic	[35]	NM_012258
HEY2	member of the hairy and enhancer of split-related (HESR) family of basic helix-loop-helix (bHLH)-type transcriptional factors	[35]	NM_012259
HEYL (HEY3)	member of the hairy and enhancer of split-related (HESR) family of basic helix-loop-helix (bHLH)-type transcription factors	[35]	NM_014571
IL3	interleukin 3; growth promoting cytokine	[36]	NM_000588
INHBA	inhibin beta A	[37]	NM_002192
MDK	mouse: promotes survival of mesencephalic neurons in culture, might have ubiquitous neurotrophic effect on DA neurons	[38]	NM_002391
MEF2C	mouse: mef2 genes appear to be associated with withdrawal of neurons from cell cycle and expression of a differentiated phenotype	[39]	NM_002397
MLL	histone methyltransferase; neuronal differentiation	[40]	NM_005933

Gene	Description	Ref	Accession
NCOA6	potential to enhance the activity of various transcription factors; could modulate chromatin structure by recruiting factors with HAT activity to the promoter (e.g., CBP/p300)	[41]	NM_014071
NDN	expressed in post-mitotic neurons; binds to ARNT2	[42]	NM_002487
NDP	Norrie disease protein; activates Wnt pathway, mutations lead to Norrie disease, expressed in the neural tube in mice	[43,44]	NM_000266
NEUROD1	proneural basic helix-loop-helix transcription factor that is essential for the development of the CNS; neuronal differentiation	[45]	NM_002500
NOG	important for specification of midbrain DA neurons and may be necessary for their long-term survival	[46]	NM_005450
NOTCH2	role in a various developmental processes, controlling cell fate decisions	[47]	NM_024408
NPTX1	synaptogenic activity; constitutively expressed in adult neurons and can form small cell surface clusters that can cocluster AMPA receptors	[48]	NM_002522
NRCAM	expressed by ventro-mesencephalic DA neurons; co-receptor for NRP2, also required for full manifestation of the repulsive effect of Sema3B and 3F	[49]	NM_005010
NRG1	major role in neural, mammary and cardiac development; axonally derived NRG1 binds to erbb2 receptors expressed on Schwann cells	[50]	NM_013957
NRP1	Sema3A receptor; neuronal cell guidance and axonal growth during the development of the nervous system	[51]	NM_003873
NRP2	Sema3F receptor; expressed in the ventral mesencephalon and ventro-mesencephalic DA neurons during development and in the adult brain	[49]	NM_003872
NTN1	axon guidance	[52]	NM_004822
ODZ1	odd Oz/ten-m homolog 1; cellular signal transducer	[53]	NM_014253
PAFAH1B1	influences microtubule function	[54]	NM_000430
PARD3 (PAR3)	part of a protein complex that includes Par6, atypical protein kinases C and the Cdc42 GTPase	[55]	NM_019619
PARD6B (PAR6B)	part of a protein complex that includes Par3, atypical protein kinases C and the Cdc42 GTPase, regulates cell polarity in different cell types.	[55,56,57]	NM_032521
PAX3	member of the paired box (PAX) family of transcription factors; neural stem cell marker	[58]	NM_181461
PAX5	member of the paired box (PAX) family of transcription factors	[59]	NM_016734
PAX6	member of the paired box (PAX) family of transcription factors; neural stem cell marker	[58,60]	NM_000280
POU3F3 (BRN1)	transcription factor, expressed in CNS	[61]	NM_006236
POU4F1 (BRN3A)	transcription factor, also found in subpopulations of DA neurons or in DA neuron region	[62]	NM_006237
PTN	pleitrophin; neurite outgrowth promotin activity	[63]	NM_002825
RAC1	promotes cell spreading, lamellopodia formation and neurite outgrowth	[64]	NM_006908
ROBO1	axon guidance; rat: in developing and adult mdDA neurons, expressed in neurons throughout the SN and VTA	[52]	NM_002941
RTN4 (NOGO)	inhibitor of neurite outgrowth; involvement in restricting the plasticity of the CNS	[65]	NM_007008

Gene	Description	Ref	Accession
S100A6	binds calcium; found in subpopulations of neurons and astrocytes	[66]	NM_014624
S100B	S100 calcium binding protein B	[67]	NM_006272
SEMA4D	semaphorin 4D; tranmembrane protein, axon guidance	[68]	NM_006378
SHH	morphogen, expressed in midbrain regions, required for proper development of mdDA neurons	[69]	NM_000193
SLIT2	axon guidance; rat: SLIT2 is expressed in adult SN neurons only	[52,69]	NM_004787
SOX8	transcription factor, regulation of embryonic development and in the determination of the cell fate	[70]	NM_014587
STAT3	signal transducer and activator of transcription 3 ; transcription factor	[71]	NM_003150
TNR	extracellular matix protein, primarily expressed in the central nervous system	[72]	NM_003285
VEGFA	isoform VEGF165 binds to both NRP1 and NRP2	[73]	NM_003376
YWHAH (14-3-3 eta)	most abundantly expressed in brain; putative functions: cell survival, signaling, cell growth, division, adhesion, differentiation, apoptosis, regulation of ion channels	[74]	NM_003405

References

1. Soreq H, Seidman S (2001) Acetylcholinesterase--new roles for an old actor. Nature reviews Neuroscience 2: 294-302.
2. Libert F, Passage E, Parmentier M, Simons MJ, Vassart G, et al. (1991) Chromosomal mapping of A1 and A2 adenosine receptors, VIP receptor, and a new subtype of serotonin receptor. Genomics 11: 225-227.
3. Iwahara T, Fujimoto J, Wen D, Cupples R, Bucay N, et al. (1997) Molecular characterization of ALK, a receptor tyrosine kinase expressed specifically in the nervous system. Oncogene 14: 439-449.
4. Kesavapany S, Banner SJ, Lau KF, Shaw CE, Miller CC, et al. (2002) Expression of the Fe65 adapter protein in adult and developing mouse brain. Neuroscience 115: 951-960.
5. Saunders AM, Strittmatter WJ, Schmechel D, George-Hyslop PH, Pericak-Vance MA, et al. (1993) Association of apolipoprotein E allele epsilon 4 with late-onset familial and sporadic Alzheimer's disease. Neurology 43: 1467-1472.
6. Drutel G, Heron A, Kathmann M, Gros C, Mace S, et al. (1999) ARNT2, a transcription factor for brain neuron survival? The European journal of neuroscience 11: 1545-1553.
7. Baloh RH, Tansey MG, Lampe PA, Fahrner TJ, Enomoto H, et al. (1998) Artemin, a novel member of the GDNF ligand family, supports peripheral and central neurons and signals through the GFRalpha3-RET receptor complex. Neuron 21: 1291-1302.
8. Villa A, Liste I, Courtois ET, Seiz EG, Ramos M, et al. (2009) Generation and properties of a new human ventral mesencephalic neural stem cell line. Experimental cell research 315: 1860-1874.
9. Mori K, Kanemura Y, Fujikawa H, Nakano A, Ikemoto H, et al. (2002) Brain-specific angiogenesis inhibitor 1 (BAI1) is expressed in human cerebral neuronal cells. Neuroscience research 43: 69-74.
10. Martin JL, Finsterwald C (2011) Cooperation between BDNF and glutamate in the regulation of synaptic transmission and neuronal development. Communicative & integrative biology 4: 14-16.
11. Di Pasquale E, Brivanlou AH (2009) Bone morphogenetic protein 15 (BMP15) acts as a BMP and Wnt inhibitor during early embryogenesis. The Journal of biological chemistry 284: 26127-26136.
12. Lu HZ, Hu JG (2009) Expression of bone morphogenetic proteins-2/4 in neural stem cells and their lineages. Acta neurobiologiae experimentalis 69: 441-447.
13. Kee K, Gonsalves JM, Clark AT, Pera RA (2006) Bone morphogenetic proteins induce germ cell differentiation from human embryonic stem cells. Stem cells and development 15: 831-837.
14. Endo R, Saito T, Asada A, Kawahara H, Ohshima T, et al. (2009) Commitment of 1-methyl-4-phenylpyrinidinium ion-induced neuronal cell death by proteasome-mediated degradation of p35 cyclin-dependent kinase 5 activator. The Journal of biological chemistry 284: 26029-26039.
15. Wang X, Ching YP, Lam WH, Qi Z, Zhang M, et al. (2000) Identification of a common protein association region in the neuronal Cdk5 activator. The Journal of biological chemistry 275: 31763-31769.
16. Gibbons AS, Scarr E, McLean C, Sundram S, Dean B (2009) Decreased muscarinic receptor binding in the frontal cortex of bipolar disorder and major depressive disorder subjects. Journal of affective disorders 116: 184-191.
17. Edman LC, Mira H, Erices A, Malmersjo S, Andersson E, et al. (2008) Alpha-chemokines regulate proliferation, neurogenesis, and dopaminergic differentiation of ventral midbrain precursors and neurospheres. Stem Cells 26: 1891-1900.
18. Feyissa AM, Chandran A, Stockmeier CA, Karolewicz B (2009) Reduced levels of NR2A and NR2B subunits of NMDA receptor and PSD-95 in the prefrontal cortex in major depression. Progress in neuro-psychopharmacology & biological psychiatry 33: 70-75.
19. Bauer M, Szulc J, Meyer M, Jensen CH, Terki TA, et al. (2008) Delta-like 1 participates in the specification of ventral midbrain progenitor derived dopaminergic neurons. Journal of neurochemistry 104: 1101-1115.
20. Beaulieu JM, Gainetdinov RR (2011) The physiology, signaling, and pharmacology of dopamine receptors. Pharmacological reviews 63: 182-217.
21. Endo Y, Beauchamp E, Woods D, Taylor WG, Toretsky JA, et al. (2008) Wnt-3a and Dickkopf-1 stimulate neurite outgrowth in Ewing tumor cells via a Frizzled3- and c-Jun N-terminal kinase-dependent mechanism. Molecular and cellular biology 28: 2368-2379.

22. McClelland AC, Sheffler-Collins SI, Kayser MS, Dalva MB (2009) Ephrin-B1 and ephrin-B2 mediate EphB-dependent presynaptic development via syntenin-1. Proceedings of the National Academy of Sciences of the United States of America 106: 20487-20492.
23. Gadadhar A, Marr R, Lazarov O (2011) Presenilin-1 regulates neural progenitor cell differentiation in the adult brain. The Journal of neuroscience : the official journal of the Society for Neuroscience 31: 2615-2623.
24. Francis YI, Diss JK, Kariti M, Stephanou A, Latchman DS (2007) p300 activation by Presenilin 1 but not by its M146L mutant. Neuroscience letters 413: 137-140.
25. Chen Y, Fu AK, Ip NY (2008) Bidirectional signaling of ErbB and Eph receptors at synapses. Neuron glia biology 4: 211-221.
26. Haedicke J, Brown C, Naghavi MH (2009) The brain-specific factor FEZ1 is a determinant of neuronal susceptibility to HIV-1 infection. Proceedings of the National Academy of Sciences of the United States of America 106: 14040-14045.
27. Greene JM, Li YL, Yourey PA, Gruber J, Carter KC, et al. (1998) Identification and characterization of a novel member of the fibroblast growth factor family. The European journal of neuroscience 10: 1911-1925.
28. Mudo G, Bonomo A, Di Liberto V, Frinchi M, Fuxe K, et al. (2009) The FGF-2/FGFRs neurotrophic system promotes neurogenesis in the adult brain. Journal of neural transmission 116: 995-1005.
29. Robertson SP (2005) Filamin A: phenotypic diversity. Current opinion in genetics & development 15: 301-307.
30. Krieglstein K (2004) Factors promoting survival of mesencephalic dopaminergic neurons. Cell and tissue research 318: 73-80.
31. Lang JC, Costa T (1989) Distribution of the alpha-subunit of the guanine nucleotide-binding protein Gi2 and its comparison to G alpha o. Journal of receptor research 9: 313-329.
32. Kim JW, Dang CV (2005) Multifaceted roles of glycolytic enzymes. Trends in biochemical sciences 30: 142-150.
33. Majdzadeh N, Wang L, Morrison BE, Bassel-Duby R, Olson EN, et al. (2008) HDAC4 inhibits cell-cycle progression and protects neurons from cell death. Developmental neurobiology 68: 1076-1092.
34. Gregoire S, Yang XJ (2005) Association with class IIa histone deacetylases upregulates the sumoylation of MEF2 transcription factors. Molecular and cellular biology 25: 2273-2287.
35. Katoh M (2007) Integrative genomic analyses on HES/HEY family: Notch-independent HES1, HES3 transcription in undifferentiated ES cells, and Notch-dependent HES1, HES5, HEY1, HEY2, HEYL transcription in fetal tissues, adult tissues, or cancer. International journal of oncology 31: 461-466.
36. Zhao B, Schwartz JP (1998) Involvement of cytokines in normal CNS development and neurological diseases: recent progress and perspectives. Journal of neuroscience research 52: 7-16.
37. Zhang SJ, Zou M, Lu L, Lau D, Ditzel DA, et al. (2009) Nuclear calcium signaling controls expression of a large gene pool: identification of a gene program for acquired neuroprotection induced by synaptic activity. PLoS genetics 5: e1000604.
38. Ohgake S, Shimizu E, Hashimoto K, Okamura N, Koike K, et al. (2009) Dopaminergic hypofunctions and prepulse inhibition deficits in mice lacking midkine. Progress in neuro-psychopharmacology & biological psychiatry 33: 541-546.
39. Lyons GE, Micales BK, Schwarz J, Martin JF, Olson EN (1995) Expression of mef2 genes in the mouse central nervous system suggests a role in neuronal maturation. The Journal of neuroscience : the official journal of the Society for Neuroscience 15: 5727-5738.
40. Lim DA, Huang YC, Swigut T, Mirick AL, Garcia-Verdugo JM, et al. (2009) Chromatin remodelling factor Mll1 is essential for neurogenesis from postnatal neural stem cells. Nature 458: 529-533.
41. Mahajan MA, Samuels HH (2008) Nuclear receptor coactivator/coregulator NCoA6(NRC) is a pleiotropic coregulator involved in transcription, cell survival, growth and development. Nuclear receptor signaling 6: e002.
42. Friedman ER, Fan CM (2007) Separate necdin domains bind ARNT2 and HIF1alpha and repress transcription. Biochemical and biophysical research communications 363: 113-118.
43. Warden SM, Andreoli CM, Mukai S (2007) The Wnt signaling pathway in familial exudative vitreoretinopathy and Norrie disease. Seminars in ophthalmology 22: 211-217.
44. Ye X, Smallwood P, Nathans J (2011) Expression of the Norrie disease gene (Ndp) in developing and adult mouse eye, ear, and brain. Gene expression patterns : GEP 11: 151-155.
45. Kuwabara T, Hsieh J, Muotri A, Yeo G, Warashina M, et al. (2009) Wnt-mediated activation of NeuroD1 and retro-elements during adult neurogenesis. Nature neuroscience 12: 1097-1105.
46. Chiba S, Lee YM, Zhou W, Freed CR (2008) Noggin enhances dopamine neuron production from human embryonic stem cells and improves behavioral outcome after transplantation into Parkinsonian rats. Stem Cells 26: 2810-2820.
47. Louvi A, Artavanis-Tsakonas S (2006) Notch signalling in vertebrate neural development. Nature reviews Neuroscience 7: 93-102.
48. Xu D, Hopf C, Reddy R, Cho RW, Guo L, et al. (2003) Narp and NP1 form heterocomplexes that function in developmental and activity-dependent synaptic plasticity. Neuron 39: 513-528.
49. Torre ER, Gutekunst CA, Gross RE (2010) Expression by midbrain dopamine neurons of Sema3A and 3F receptors is associated with chemorepulsion in vitro but a mild in vivo phenotype. Molecular and cellular neurosciences 44: 135-153.
50. Fricker FR, Zhu N, Tsantoulas C, Abrahamsen B, Nassar MA, et al. (2009) Sensory axon-derived neuregulin-1 is required for axoglial signaling and normal sensory function but not for long-term axon maintenance. The Journal of neuroscience : the official journal of the Society for Neuroscience 29: 7667-7678.
51. Hermanson E, Borgius L, Bergsland M, Joodmardi E, Perlmann T (2006) Neuropilin1 is a direct downstream target of Nurr1 in the developing brain stem. Journal of neurochemistry 97: 1403-1411.
52. Smidt MP, Burbach JP (2007) How to make a mesodiencephalic dopaminergic neuron. Nature reviews Neuroscience 8: 21-32.
53. Ben-Zur T, Feige E, Motro B, Wides R (2000) The mammalian Odz gene family: homologs of a Drosophila pair-rule gene with expression implying distinct yet overlapping developmental roles. Developmental biology 217: 107-120.
54. Keays DA (2007) Neuronal migration: unraveling the molecular pathway with humans, mice, and a fungus. Mammalian genome : official journal of the International Mammalian Genome Society 18: 425-430.
55. Gao L, Macara IG, Joberty G (2002) Multiple splice variants of Par3 and of a novel related gene, Par3L, produce proteins with different binding properties. Gene 294: 99-107.
56. Alarcon VB (2010) Cell polarity regulator PARD6B is essential for trophectoderm formation in the preimplantation mouse embryo. Biology of reproduction 83: 347-358.
57. Durgan J, Kaji N, Jin D, Hall A (2011) Par6B and Atypical PKC Regulate Mitotic Spindle Orientation during Epithelial Morphogenesis. The Journal of biological chemistry 286: 12461-12474.
58. Kuegler PB, Zimmer B, Waldmann T, Baudis B, Ilmjarv S, et al. (2010) Markers of murine embryonic and neural stem cells, neurons and astrocytes: reference points for developmental neurotoxicity testing. ALTEX 27: 17-42.
59. Simon HH, Bhatt L, Gherbassi D, Sgado P, Alberi L (2003) Midbrain dopaminergic neurons: determination of their developmental fate by transcription factors. Annals of the New York Academy of Sciences 991: 36-47.
60. Li XJ, Du ZW, Zarnowska ED, Pankratz M, Hansen LO, et al. (2005) Specification of motoneurons from human embryonic stem cells. Nature biotechnology 23: 215-221.
61. Jin Z, Liu L, Bian W, Chen Y, Xu G, et al. (2009) Different transcription factors regulate nestin gene expression during P19 cell neural differentiation and central nervous system development. The Journal of biological chemistry 284: 8160-8173.
62. Nelander J, Hebsgaard JB, Parmar M (2009) Organization of the human embryonic ventral mesencephalon. Gene expression patterns : GEP 9: 555-561.

63. Landgraf P, Wahle P, Pape HC, Gundelfinger ED, Kreutz MR (2008) The survival-promoting peptide Y-P30 enhances binding of pleiotrophin to syndecan-2 and -3 and supports its neuritogenic activity. The Journal of biological chemistry 283: 25036-25045.
64. Hajdo-Milasinovic A, van der Kammen RA, Moneva Z, Collard JG (2009) Rac3 inhibits adhesion and differentiation of neuronal cells by modifying GIT1 downstream signaling. Journal of cell science 122: 2127-2136.
65. Oertle T, Schwab ME (2003) Nogo and its paRTNers. Trends in cell biology 13: 187-194.
66. Yamashita N, Ilg EC, Schafer BW, Heizmann CW, Kosaka T (1999) Distribution of a specific calcium-binding protein of the S100 protein family, S100A6 (calcyclin), in subpopulations of neurons and glial cells of the adult rat nervous system. The Journal of comparative neurology 404: 235-257.
67. Villarreal A, Aviles Reyes RX, Angelo MF, Reines AG, Ramos AJ (2011) S100B alters neuronal survival and dendrite extension via RAGE-mediated NF-kappaB signaling. Journal of neurochemistry 117: 321-332.
68. Vodrazka P, Korostylev A, Hirschberg A, Swiercz JM, Worzfeld T, et al. (2009) The semaphorin 4D-plexin-B signalling complex regulates dendritic and axonal complexity in developing neurons via diverse pathways. The European journal of neuroscience 30: 1193-1208.
69. Van den Heuvel DM, Pasterkamp RJ (2008) Getting connected in the dopamine system. Progress in neurobiology 85: 75-93.
70. Stolt CC, Wegner M (2010) SoxE function in vertebrate nervous system development. The international journal of biochemistry & cell biology 42: 437-440.
71. Snyder M, Huang XY, Zhang JJ (2011) Stat3 is essential for neuronal differentiation through direct transcriptional regulation of the Sox6 gene. FEBS letters 585: 148-152.
72. El Ayachi I, Fernandez C, Baeza N, De Paula AM, Pesheva P, et al. (2011) Spatiotemporal distribution of tenascin-R in the developing human cerebral cortex parallels neuronal migration. The Journal of comparative neurology 519: 2379-2389.
73. Pellet-Many C, Frankel P, Jia H, Zachary I (2008) Neuropilins: structure, function and role in disease. The Biochemical journal 411: 211-226.
74. Anantharam V, Lehrmann E, Kanthasamy A, Yang Y, Banerjee P, et al. (2007) Microarray analysis of oxidative stress regulated genes in mesencephalic dopaminergic neuronal cells: relevance to oxidative damage in Parkinson's disease. Neurochemistry international 50: 834-847.
