# Supplementary material for: Extensive Transcriptional Regulation of Chromatin Modifiers during Human Neurodevelopment
Source: PLoS One. 2012 May 9;7(5):e36708. doi: 10.1371/journal.pone.0036708 (PMC3348879; doi:10.1371/journal.pone.0036708)
Supplement: Figure S3 — Expression of neuro-developmental genes in NEP and NCP. hESC were differentiated into NEP or NCP. RNA was prepared from all types and qPCR was performed using primers specific for the indicated neurodevelopmental regulator genes. Threshold cycle values (Ct) were measured with a Biorad light cycler. Ct values were normalized to house keeping genes, and relative gene expressions were calculated by normalization to hESC expression levels. Data are means of three independent differentiations +/− standart deviation (SD). p-values were calculated with Studens t-test and corrected for false discovery rate (FDR) according to Benjamini-Hochberg. They correspond to the statistical difference from the expression levels in hESC. Data corresponds to Fig. 4C. (PDF) [file pone.0036708.s003.pdf]

## Figure S3: Expression of neuro-developmental genes in NEP and NCP

| Gene | rel. Expression NEP | SD | FDR corr. p-value | rel. Expression NCP | SD | FDR corr.p-value |
|---|---|---|---|---|---|---|
| ACHE | 2.16 | 0.73 | 0.07 | 0.75 | 0.11 | 0.19 |
| ADORA1 | 3.26 | 3.98 | 0.38 | 1.74 | 0.50 | 0.11 |
| ADORA2A | 1.31 | 0.17 | 0.06 | 0.16 | 0.08 | 0.04 |
| ALK | 22.79 | 4.33 | 0.04 | 11.72 | 7.07 | 0.04 |
| APBB1 | 2.83 | 0.62 | 0.02 | 1.77 | 0.15 | 0.05 |
| APOE | 0.93 | 0.20 | 0.64 | 0.10 | 0.04 | 0.02 |
| ARNT2 | 6.95 | 1.71 | 0.02 | 4.05 | 0.51 | 0.04 |
| ARTN | 0.40 | 0.03 | 0.02 | 0.05 | 0.02 | 0.02 |
| ASCL1 | 5.70 | 3.70 | 0.10 | 0.06 | 0.01 | 0.02 |
| BAI1 | 2.42 | 0.83 | 0.04 | 1.18 | 0.72 | 0.94 |
| BDNF | 1.05 | 0.46 | 0.85 | 9.17 | 1.70 | 0.04 |
| BMP15 | 5.08 | 3.15 | 0.03 | 2.52 | 2.63 | 0.66 |
| BMP2 | 1.67 | 0.66 | 0.34 | 0.15 | 0.03 | 0.04 |
| BMP4 | 9.99 | 6.77 | 0.04 | 3.21 | 1.19 | 0.04 |
| BMP8B | 6.53 | 2.29 | 0.03 | 1.04 | 0.88 | 0.73 |
| CDK5R1 | 1.88 | 0.53 | 0.06 | 0.45 | 0.04 | 0.02 |
| CDK5RAP1 | 2.50 | 0.98 | 0.07 | 0.75 | 0.11 | 0.31 |
| CDK5RAP2 | 3.22 | 0.18 | 0.02 | 1.92 | 0.21 | 0.02 |
| CDK5RAP3 | 3.12 | 1.27 | 0.04 | 1.12 | 0.23 | 0.57 |
| CHRM2 | 1.60 | 0.36 | 0.69 | 2.63 | 0.95 | 0.23 |
| CXCL1 | 0.40 | 0.19 | 0.05 | 0.07 | 0.02 | 0.01 |
| DLG4 | 2.98 | 0.74 | 0.03 | 2.83 | 0.62 | 0.03 |
| DLL1 | 30.58 | 17.04 | 0.02 | 0.04 | 0.02 | 0.02 |
| DRD1 | 1.17 | 0.25 | 0.78 | 0.93 | 0.26 | 0.89 |
| DRD2 | 16.90 | 15.67 | 0.15 | 0.54 | 0.26 | 0.12 |
| DVL3 | 2.38 | 0.56 | 0.04 | 2.19 | 0.37 | 0.03 |
| EFNB1 | 15.00 | 4.39 | 0.02 | 37.46 | 7.80 | 0.00 |
| EGF | 8.15 | 5.26 | 0.09 | 1.22 | 0.25 | 0.71 |
| EP300 | 1.85 | 0.40 | 0.04 | 0.97 | 0.14 | 0.80 |
| ERBB2 | 0.90 | 0.09 | 0.47 | 0.97 | 0.18 | 0.81 |
| FEZ1 | 2.12 | 1.44 | 0.26 | 1.01 | 0.10 | 0.97 |
| FGF13 | 1.88 | 0.05 | 0.04 | 0.48 | 0.22 | 0.07 |
| FGF2 | 0.64 | 0.21 | 0.05 | 0.13 | 0.03 | 0.02 |
| FLNA | 1.32 | 0.42 | 0.29 | 1.80 | 0.38 | 0.04 |
| GDNF | 3.17 | 1.28 | 0.06 | 0.57 | 0.23 | 0.19 |
| GNAO1 | 0.49 | 0.09 | 0.02 | 0.02 | 0.01 | 0.02 |
| GPI | 0.55 | 0.07 | 0.09 | 0.48 | 0.13 | 0.08 |
| GRIN1 | 0.57 | 0.19 | 0.07 | 0.04 | 0.04 | 0.04 |
| HDAC4 | 1.94 | 0.44 | 0.04 | 0.94 | 0.20 | 0.62 |
| HDAC7 | 1.54 | 0.48 | 0.77 | 2.28 | 0.34 | 0.25 |
| HES1 | 1.24 | 0.66 | 0.57 | 0.48 | 0.24 | 0.11 |
| HEY1 | 16.03 | 3.31 | 0.01 | 0.34 | 0.18 | 0.08 |
| HEY2 | 0.79 | 0.80 | 0.68 | 0.11 | 0.08 | 0.42 |
| HEYL | 6.08 | 2.58 | 0.04 | 0.59 | 0.16 | 0.05 |
| IL3 | 1.05 | 0.21 | 0.68 | 0.85 | 0.27 | 0.96 |
| INHBA | 0.53 | 0.15 | 0.18 | 1.87 | 0.66 | 0.24 |
| MDK | 4.29 | 2.09 | 0.04 | 1.01 | 0.28 | 0.04 |

| | | | | | | |
|---|---|---|---|---|---|---|
| MEF2C | 1.05 | 0.20 | 0.92 | 1.65 | 0.69 | 0.92 |
| MLL | 2.88 | 1.26 | 0.07 | 1.13 | 0.13 | 0.10 |
| NCOA6 | 9.21 | 0.84 | 0.00 | 6.65 | 1.13 | 0.00 |
| NDN | 5.17 | 0.70 | 0.04 | 11.45 | 3.61 | 0.04 |
| NDP | 12.29 | 5.03 | 0.02 | 1.86 | 1.74 | 0.06 |
| NEUROD1 | 279.50 | 200.08 | 0.02 | 0.09 | 0.02 | 0.02 |
| NOG | 12.88 | 11.90 | 0.16 | 0.45 | 0.14 | 0.06 |
| NOTCH2 | 5.37 | 2.55 | 0.04 | 7.16 | 1.44 | 0.04 |
| NPTX1 | 0.09 | 0.01 | 0.02 | 0.02 | 0.01 | 0.02 |
| NRCAM | 10.54 | 4.55 | 0.03 | 3.50 | 2.03 | 0.03 |
| NRG1 | 9.75 | 0.16 | 0.04 | 1.05 | 0.33 | 0.04 |
| NRP1 | 5.43 | 3.03 | 0.03 | 54.04 | 1.13 | 0.04 |
| NRP2 | 5.52 | 3.71 | 0.04 | 1.87 | 0.26 | 0.04 |
| NTN1 | 2.63 | 0.68 | 0.06 | 1.83 | 0.32 | 0.13 |
| ODZ1 | 2.89 | 2.19 | 0.25 | 7.38 | 2.54 | 0.19 |
| PAFAH1B1 | 2.83 | 0.85 | 0.04 | 1.58 | 0.04 | 0.04 |
| PARD3 | 1.29 | 0.20 | 0.10 | 1.49 | 0.35 | 0.14 |
| PARD6B | 3.61 | 1.98 | 0.09 | 0.01 | 0.00 | 0.06 |
| PAX3 | 180.59 | 88.11 | 0.01 | 26.65 | 4.39 | 0.01 |
| PAX5 | 1.15 | 0.26 | 1.00 | 0.83 | 0.44 | 0.78 |
| PAX6 | 562.49 | 264.06 | 0.01 | 0.74 | 0.39 | 0.02 |
| POU3F3 | 9.91 | 3.39 | 0.02 | 6.65 | 2.77 | 0.02 |
| POU4F1 | 9.86 | 13.78 | 0.33 | 0.05 | 0.02 | 0.24 |
| PTN | 4.57 | 1.18 | 0.02 | 83.14 | 39.00 | 0.02 |
| RAC1 | 2.10 | 0.42 | 0.04 | 2.14 | 0.18 | 0.04 |
| ROBO1 | 3.11 | 0.73 | 0.03 | 11.41 | 2.25 | 0.03 |
| RTN4 | 1.12 | 0.27 | 0.50 | 1.46 | 0.60 | 0.62 |
| S100A6 | 6.00 | 4.19 | 0.11 | 1.46 | 0.36 | 0.06 |
| S100B | 1.31 | 0.18 | 0.91 | 30.76 | 19.64 | 0.62 |
| SEMA4D | 1.18 | 0.20 | 0.27 | 0.05 | 0.01 | 0.28 |
| SHH | 4.60 | 5.44 | 0.46 | 0.47 | 0.06 | 0.54 |
| SLIT2 | 59.68 | 45.12 | 0.02 | 46.89 | 8.97 | 0.02 |
| SOX8 | 1.54 | 0.39 | 0.09 | 1.95 | 0.74 | 0.13 |
| STAT3 | 1.68 | 0.61 | 0.13 | 2.57 | 0.26 | 0.20 |
| TNR | 1.21 | 0.46 | 0.63 | 0.82 | 0.33 | 0.88 |
| VEGFA | 2.25 | 1.92 | 0.33 | 2.43 | 0.57 | 0.43 |
| YWHAH | 1.01 | 0.32 | 0.93 | 0.69 | 0.25 | 0.92 |
