# Supplementary material for: Extensive Transcriptional Regulation of Chromatin Modifiers during Human Neurodevelopment
Source: PLoS One. 2012 May 9;7(5):e36708. doi: 10.1371/journal.pone.0036708 (PMC3348879; doi:10.1371/journal.pone.0036708)
Supplement: Figure S4 — 150 Epigenetic regulators examined in this study. The epigenetic regulator genes investigated in this study by qPCR are listed alphabetically by their gene symbol. The cDNAs are defined by the respective RefSeq accession numbers (as used in NCBI data bases). Alternative gene names frequently used in the literature have been added in paretheses. The references provide further background information. (PDF) [file pone.0036708.s004.pdf]

Figure S4: 150 epigenetic regulators examined in this study

Gene	Description	Ref	Accession No.
ARID1A (BAF250a)	defining subunit of the BAF co-repressor complex	[1]	NM_006015
ASH1L	methylation of H3K4 and / or K36	[2,3]	NM_018489
ATF2	CRE binding protein, component of Tip60 HAT complex	[4]	NM_001880
AURKA	phosphorylation of H3S10, role in mitosis	[5]	NM_003600
AURKB	phosphorylation of H3S10, role in mitosis	[5]	NM_004217
AURKC	phosphorylation of H3S10, role in mitosis	[6]	NM_003160
BAF45A (PHF10)	component of neural progenitor BAF complex	[7]	NM_133325
BAF53A	component of neural progenitor BAF complex	[7]	NM_004301
BAF53B	component of neuron BAF complex	[7]	NM_016188
BAF60A (SMARCD1)	general component of BAF complex	[7]	NM_003076
BAF60C (SMARCD3)	component of neural progenitor and neuron BAF complex	[7]	NM_003078
BAZ1A (hACF1)	component of remodeling complexes ACF, CHRAC, WICH	[8]	NM_182648
BAZ1B (WSTF)	subunit of chromatin remodeling complexes WHICH and WINAC	[8]	NM_032408
BAZ2A	bromo domain protein, component of NoRC complex	[9]	NM_013450
BAZ2B	bromodomain protein, component of NoRC complex	[10]	NM_013450
BMI1	component of PRC1, required for self-renewal of neural and neural crest stem cells in mouse	[11]	NM_005180
BPTF (NURF301)	H3K4me binding, specific component of NURF complex	[12]	NM_182641
BRD1	bromodomain protein, component of MOZ/MORF complex, expressed in brain	[13]	NM_014577
BRD2	bromodomain protein, binds to H3K12Ac, H4K5Ac, H4K12Ac	[14]	NM_005104
BRD3	bromodomain protein, binds to H3K12Ac, H4K5Ac, H4K12Ac	[15]	NM_007371
BRD4	bromodomain protein, binds to H3K12Ac, H4K5Ac, H4K12Ac	[14]	NM_014299
BRD7	bromodomain protein binds to H3K14Ac defining subunit of PBAF complex	[1]	NM_013263
BRD8	bromo domain protein, component of Tip60 HAT complex	[16]	NM_183359
BRPF1	component of MOZ/MORF complex, stimulates HAT activity	[13,17]	NM_004634
BRPF3	component of MOZ/MORF complex	[13]	NM_015695

Gene	**Description**	**Ref**	**Accession No.**
BRWD1	bromo domain protein	[18]	NM_018963
CARM1 (PRMT4)	type I arginine methyl transferase, methylates H3R17 and H3R26, transcriptional activation	[19]	NM_199141
CBX1 (HP1β)	binds to H3K9me3, formation of heterochromatin	[20]	NM_006807
CBX3 (HP1γ)	binds to H3K9me3, located in heterochromatin as well as euchromatin, role in gene silencing in euchromatin	[20]	NM_007276
CBX4 (Pc2)	component of PRC1, binding to H3K27me3 and K9me3	[21,22]	NM_003655
CBX5 (HP1α)	chromodomain protein, located exclusively in heterochromatin	[23]	NM_012117
CBX6	chromodomain protein, but no binding to methylated histones is described	[24]	NM_014292
CBX7	chromodomain protein, Pc hommologe, binds to H3K9me and H3K27me, interacts with Ring1b	[25]	NM_175709
CBX8	component of PRC1, binds to H3K37me3	[11]	NM_020649
CDYL	chromodomain protein, involved in transcriptional repression as a component of the REST complex, not able to bind methylated lysines	[26]	NM_004824
CDYL2	chromodomain protein, binds to H3K9me3, H1.4K26me3	[26]	NM_152342
CHD1	chromodomain protein, ATPase, subunit of CHD1, binds to H3K4me1, 2 and 3	[8]	NM_001270
CHD2	chromodomain protein, expressed in forebrain in mouse	[27]	NM_001271
CHD3 (Mi-2a)	chromodomain protein, ATPase, subunit of NuRD	[8]	NM_001005273
CHD4 (Mi-2b)	chromodomain protein, ATPase, subunit of NuRD, interaction partner of NAB2	[8]	NM_001273
CHD5	chromodomain protein, similar to CHD3 and CHD4, expressed in neural tissue and neurons but not in glia cells	[28,29]	NM_015557
CHD6	chromodomain protein, might have a role in motor coordination	[30]	NM_032221
CHD7	chromodomain protein, binds to H3K4me, role in neural crest formation	[31]	NM_017780
CHD8	chromodomain protein, involved in insulation of heterochromatin	[8,32]	NM_020920
CHD9	chromodomain protein, poorly characterised, ATPase activity	[32]	NM_025134
CSRP2BP (ATAC2)	acetylation of H3 and H4	[33]	NM_177926
CTBP1	inhibition of transcription by binding to p300 and / or by complex formation with HDAC1/2	[34,35]	NM_001328
CTBP2	in complex with CTBP1, k.o. mice defects in neuronal development	[36]	NM_022802
CTCF	insulator protein, organisation of chromatin territories	[37]	NM_006565
DNMT1	CpG methylation, maintenance of DNA methylation status	[38]	NM_001379
DNMT3A	de novo DNA methylation	[38]	NM_022552

Gene	Description	Ref	Accession No.
DNMT3B	de novo DNA methylation, hESC stem cell marker	[38,39]	NM_006892
DOT1L (KMT4)	methylation of H3K79	[40]	NM_032482
DZIP3	ubiquitination of H2AK119, blocks transcriptional elongation	[41]	NM_0146648
EED	component of PRC2	[11]	NM_003797
EHMT2 (G9a, KMT1C)	methylation of H3K9me1 and me2 in euchromatic regions	[40]	NM_006709
ESCO1	component of cohesin, slight acetylation activity towards non-histone proteins, repression of transcription via interaction with LSD1 in yeast	[42]	NM_052911
ESCO2	component of cohesin, repression of transcription via interaction with CoREST and G9a in yeast	[42]	NM_001017420
EZH1	methylation of H3K27me3, component of PRC2, expressed in adult tissue and able to compact chromatin	[43,44]	NM_001991
EZH2	methylation of H3K27me3, component of PRC2	[11]	NM_004456
HAT1	acetylation of H3K5 and H3K12	[45]	NM_003642
HDAC1	histone deacetylation, class I HDAC, component of BHC complex, involved in repression of neuronal genes	[46,47]	NM_004964
HDAC10	histone deacetylation, class II HDAC	[46]	NM_032019
HDAC11	histone deacetylation, class IV HDAC	[46]	NM_024827
HDAC2	histone deacetylation, class II HDAC,	[46]	NM_001527
HDAC3	histone deacetylation, class I HDAC,	[46]	NM_003883
HDAC4	histone deacetylation, class II HDAC, highly expressed in brain	[46]	NM_006037
HDAC5	histone deacetylation, class II HDAC	[46]	NM_005474
HDAC6	histone deacetylation, class II HDAC	[46]	NM_006044
HDAC7	histone deacetylation, class II HDAC	[46]	NM_016596
HDAC8	histone deacetylation, class I HDAC	[46]	NM_018486
HDAC9	histone deacetylation, class III HDAC	[46]	NM_178425
ING1	PHD domain, binds to H3K4me, binds to Phosphatidylinositol, associated with p300 and HDAC1/2	[48]	NM_001537
ING2	PHD domain, binds to H3K4me, binds to Phosphatidylinositol, associated with p300 and HDAC1/2	[48]	NM_001564
ING3	component of Tip60 HAT complex, binding to H3K4me via PHD domain,	[48,49]	NM_198267
ING4	PHD domain, binds to H3K4me	[48]	NM_016162
ING5	binds to H3K4me, PHD containing protein, component of HBO1 and MOZ/MORF complex	[50]	NM_032329

Gene	Description	Ref	Accession No.
INO80	ATPase subunit of INO80	[8]	NM_017553
KAT2A (GCN5)	acetylation of multiple lysines in H3, component of GCN5 complex	[45]	NM_021078
KAT2B	acetylation of H3K9, K14, K18	[40,45]	NM_003884
KAT5 (Tip60)	histone acetylation, several K residues	[45]	NM_006388
KDM1 (LSD1)	Demethylation of H3K4 and K9 me2 and me1, regulates terminal differentiation	[51,52]	NM_015013
KDM4A	Demethylation of H3K9me3/2 and H3K36me3/2	[51]	NM_014663
KDM4C	Demethylation of H3K9/K36me2 and me3	[51]	NM_015061
KDM5B	Demethylation of H3K4me2 and me3,	[51]	NM_006618
KDM5C	Demethylation of H3K4me2 and me3, increased expression in mouse brain	[51]	NM_004187
KDM6B	Demethylation of H3K27me, required for early neural differentiation	[51,53]	NM_001080424
MBD1	methyl CpG binding, involved in HP1 mediated heterochromatin formation	[38]	NM_015844
MBD2	methyl CpG binding, component of NuRD complex, involved in Oct4 silencing	[38]	NM_003927
MBD3	CpG methyl binding protein, component of NuRD complex	[8,38]	NM_003926
MBD4	methyl-CpG binding protein	[38]	NM_003925
MECP2	methyl CpG binding, represses BDNF promotor and is released upon depolarisation of neurons	[38]	NM_004992
MLL	methylation of H3K4	[40]	NM_005933
MLL3	methylation of H3K4	[40]	NM_170606
MLL5 (KMT2E)	methylation of H3K4me1 and me2	[40]	NM_182931
MTA1	subunit of chromatin remodeling complex NURD	[8]	NM_004689
MTA2	subunit of chromatin remodeling complex NURD	[8]	NM_004739
MYSM1	Deubiquitination of H2A	[54]	NM_001085487
MYST1 (MOF)	acetylation of H4K16	[45]	NM_032188
MYST2 (MOZ, KAT7)	acetylation of H4K5, K8 and K12, role in cell cycle progression	[45]	NM_007067
MYST3 (MOZ, KAT6A)	acetylation of H3K14	[55]	NM_006766
MYST4	acetylation of H3K14	[55]	NM_012330
NCOA1 (KAT13A)	H3K9 acetylation, up-regulated neuronal commited cells in mice (not in NSC), nuclear receptor coactivator	[45,56]	NM_003743

Gene	**Description**	**Ref**	**Accession No.**
NCOA3 (KAT13B, ACTR)	acetylation, nuclear receptor coactivator,	[45,57]	NM_181659
NEK6	kinase, expressed in PNS and CNS neurons	[58]	NM_014397
NSD1 (KMT3B)	methylation of H4K20 and H3K36	[40]	NM_022455
PAK1	p21-activated kinase, expressed in brain	[59]	NM_002576
PBRM1	targeting unit of PBAF complex, defining subunit	[8,60]	NM_018165
PCGF1	component of PRC1	[11]	NM_032673
PCGF2 (Mel18)	component of PRC1	[11]	NM_007144
PHC1	polyhomeotic homolog, component of PRC1	[11]	NM_004426
PHC2	component of PRC1, mediates Hox gene expression together with PHC1	[11]	NM_198040
PHF1	PHD finger protein, binds to PRC2 and stimulates EZH2	[11]	NM_002636
PHF13 (SPOC1)	PHD finger protein, binds to H3K9me3 and H3K36me3, involved in chromatin condensation	[61]	NM_153812
PHF2	component of PRC1, mediates Hox gene expression together with PHC1	[11]	NM_198040
PHF21A (BHC80)	PHD finger protein, inhibits LSD1	[62]	NM_016621
PRMT1	type I arginine methyl transferase, methylates H4R3 and H2AR3 besides many non histone subtrates.	[19]	NM_001536
PRMT2	type I arginine methyl transferase, no methyl-transferase activity shown yet	[19]	NM_001535
PRMT3	type I arginine methyl transferase, located in cytoplasma	[19]	NM_005788
PRMT5	type II arginine methyl transferase, methylates H4R3 and H2AR3	[19]	NM_006109
PRMT6	type I arginine methyl transferase, methylates HR2me	[19,63]	NM_018137
PRMT7	type II arginine methyl transferase, methylates H4R3 and H2AR3	[19]	NM_019023
PRMT8	Arginine methylation, brain specific in mouse	[19]	NM_019854
RING1 (RING1A)	component of PRC1, stimulates H2AK119ub activity of RING1B	[11,64]	NM_002931
RNF2 (RING1B)	component of PRC1, ubiquitination of H2AK119	[11]	NM_007212
RNF20	ubiquitination of H2BK120	[65]	NM_019592
RPS6KA3	H3S10 phosphorylation	[66]	NM_004586
RPS6KA5	H3S10 phosphorylation, stress induced	[6]	NM_004755
SETD1A (SET1)	methylation of H3K4	[40]	NM_014712

Gene	Description	Ref	Accession No.
SETD1B (KMT2G)	methylation of H3K4	[40]	XM_037523
SETD2	SET domain protein, methylation of H3K36	[67]	NM_014159
SETD7 (Set7/9, KMT7)	methylation of H3K4	[40]	NM_030648
SETD8	methylation of H4K20me1, required for cell cycle progression	[40,68]	NM_020382
SETDB1 (ESET, KMT1B)	methylation of H3K9me2 and me3, involved in glutamat receptor expression	[40,69]	NM_012432
SETDB2 (KMT1F)	methylation of H3K9, inhibits dorsal regulator FGF8	[40,70]	NM_031915
SMARCA2	ATPase subunit of hBAF complex	[8]	NM_003070
SMARCA4	ATPase subunit of hBAF and PBAF complexes	[8]	NM_003072
SMYD3	methylation of H3K4	[71]	NM_022743
SPEN	associated with Co-REST complex	[72]	NM_015001
SUV39H1	methylation of H3K9me3	[40]	NM_003173
SUV420H1	methylation of H4K20me3	[40]	NM_016028
SUZ12	component of PRC2	[11]	NM_015355
TET1	methylation of hydroxy methyl cytosin	[70]	NM_030625
TET2	methylation of hydroxy methyl cytosin	[70]	NM_001127208
UBE2A (RAD6A)	ubiquitination of H2BK120, transcriptional activation	[73]	NM_003336
UBE2B (RAD6B)	ubiquitination of H2BK120	[73]	NM_003337
USP16	deubiquitination of H2A	[54]	NM_006447
USP21	deubiquitination of H2A	[54]	NM_012475
USP22	deubiquitination of H2B	[74]	NM_015276
WHSC1 (NSD2, MMSET)	methylation of H4K20 and/or H3K36	([75,76]	NM_007331

References

1. Kaeser MD, Aslanian A, Dong MQ, Yates JR, 3rd, Emerson BM (2008) BRD7, a novel PBAF-specific SWI/SNF subunit, is required for target gene activation and repression in embryonic stem cells. J Biol Chem 283: 32254-32263.
2. Gregory GD, Vakoc CR, Rozovskaia T, Zheng X, Patel S, et al. (2007) Mammalian ASH1L is a histone methyltransferase that occupies the transcribed region of active genes. Mol Cell Biol 27: 8466-8479.
3. Tanaka Y, Katagiri Z, Kawahashi K, Kioussis D, Kitajima S (2007) Trithorax-group protein ASH1 methylates histone H3 lysine 36. Gene 397: 161-168.
4. Bhoumik A, Ronai Z (2008) ATF2: a transcription factor that elicits oncogenic or tumor suppressor activities. Cell Cycle 7: 2341-2345.
5. Pascreau G, Arlot-Bonnemains Y, Prigent C (2003) Phosphorylation of histone and histone-like proteins by aurora kinases during mitosis. Prog Cell Cycle Res 5: 369-374.

6. Nowak SJ, Corces VG (2004) Phosphorylation of histone H3: a balancing act between chromosome condensation and transcriptional activation. Trends Genet 20: 214-220.
7. Lessard J, Wu JI, Ranish JA, Wan M, Winslow MM, et al. (2007) An essential switch in subunit composition of a chromatin remodeling complex during neural development. Neuron 55: 201-215.
8. Clapier CR, Cairns BR (2009) The biology of chromatin remodeling complexes. Annu Rev Biochem 78: 273-304.
9. Zhou Y, Grummt I (2005) The PHD finger/bromodomain of NoRC interacts with acetylated histone H4K16 and is sufficient for rDNA silencing. Curr Biol 15: 1434-1438.
10. Grummt I (2007) Different epigenetic layers engage in complex crosstalk to define the epigenetic state of mammalian rRNA genes. Hum Mol Genet 16 Spec No 1: R21-27.
11. Simon JA, Kingston RE (2009) Mechanisms of polycomb gene silencing: knowns and unknowns. Nat Rev Mol Cell Biol 10: 697-708.
12. Xiao H, Sandaltzopoulos R, Wang HM, Hamiche A, Ranallo R, et al. (2001) Dual functions of largest NURF subunit NURF301 in nucleosome sliding and transcription factor interactions. Mol Cell 8: 531-543.
13. Ullah M, Pelletier N, Xiao L, Zhao SP, Wang K, et al. (2008) Molecular architecture of quartet MOZ/MORF histone acetyltransferase complexes. Mol Cell Biol 28: 6828-6843.
14. Shang E, Salazar G, Crowley TE, Wang X, Lopez RA, et al. (2004) Identification of unique, differentiation stage-specific patterns of expression of the bromodomain-containing genes Brd2, Brd3, Brd4, and Brdt in the mouse testis. Gene Expr Patterns 4: 513-519.
15. LeRoy G, Rickards B, Flint SJ (2008) The double bromodomain proteins Brd2 and Brd3 couple histone acetylation to transcription. Mol Cell 30: 51-60.
16. Doyon Y, Cayrou C, Ullah M, Landry AJ, Cote V, et al. (2006) ING tumor suppressor proteins are critical regulators of chromatin acetylation required for genome expression and perpetuation. Mol Cell 21: 51-64.
17. Laue K, Daujat S, Crump JG, Plaster N, Roehl HH, et al. (2008) The multidomain protein Brpf1 binds histones and is required for Hox gene expression and segmental identity. Development 135: 1935-1946.
18. Philipps DL, Wigglesworth K, Hartford SA, Sun F, Pattabiraman S, et al. (2008) The dual bromodomain and WD repeat-containing mouse protein BRWD1 is required for normal spermiogenesis and the oocyte-embryo transition. Dev Biol 317: 72-82.
19. Di Lorenzo A, Bedford MT (2011) Histone arginine methylation. FEBS letters 585: 2024-2031.
20. Kwon SH, Workman JL (2008) The heterochromatin protein 1 (HP1) family: put away a bias toward HP1. Mol Cells 26: 217-227.
21. Ruddock-D'Cruz NT, Prashadkumar S, Wilson KJ, Heffernan C, Cooney MA, et al. (2008) Dynamic changes in localization of Chromobox (Cbx) family members during the maternal to embryonic transition. Mol Reprod Dev 75: 477-488.
22. Vincenz C, Kerppola TK (2008) Different polycomb group CBX family proteins associate with distinct regions of chromatin using nonhomologous protein sequences. Proc Natl Acad Sci U S A 105: 16572-16577.
23. Bartova E, Krejci J, Harnicarova A, Galiova G, Kozubek S (2008) Histone modifications and nuclear architecture: a review. J Histochem Cytochem 56: 711-721.
24. Ren X, Vincenz C, Kerppola TK (2008) Changes in the distributions and dynamics of polycomb repressive complexes during embryonic stem cell differentiation. Mol Cell Biol 28: 2884-2895.
25. Bernstein E, Duncan EM, Masui O, Gil J, Heard E, et al. (2006) Mouse polycomb proteins bind differentially to methylated histone H3 and RNA and are enriched in facultative heterochromatin. Mol Cell Biol 26: 2560-2569.
26. Fischle W, Franz H, Jacobs SA, Allis CD, Khorasanizadeh S (2008) Specificity of the chromodomain Y chromosome family of chromodomains for lysine-methylated ARK(S/T) motifs. J Biol Chem 283: 19626-19635.
27. Kulkarni S, Nagarajan P, Wall J, Donovan DJ, Donell RL, et al. (2008) Disruption of chromodomain helicase DNA binding protein 2 (CHD2) causes scoliosis. Am J Med Genet A 146A: 1117-1127.
28. Garcia I, Mayol G, Rodriguez E, Sunol M, Gershon TR, et al. (2010) Expression of the neuron-specific protein CHD5 is an independent marker of outcome in neuroblastoma. Molecular cancer 9: 277.
29. Thompson PM, Gotoh T, Kok M, White PS, Brodeur GM (2003) CHD5, a new member of the chromodomain gene family, is preferentially expressed in the nervous system. Oncogene 22: 1002-1011.
30. Lutz T, Stoger R, Nieto A (2006) CHD6 is a DNA-dependent ATPase and localizes at nuclear sites of mRNA synthesis. FEBS Lett 580: 5851-5857.
31. Bajpai R, Chen DA, Rada-Iglesias A, Zhang J, Xiong Y, et al. CHD7 cooperates with PBAF to control multipotent neural crest formation. Nature 463: 958-962.
32. Marfella CG, Imbalzano AN (2007) The Chd family of chromatin remodelers. Mutat Res 618: 30-40.
33. Guelman S, Kozuka K, Mao Y, Pham V, Solloway MJ, et al. (2009) The double-histone-acetyltransferase complex ATAC is essential for mammalian development. Molecular and cellular biology 29: 1176-1188.
34. Kim JH, Cho EJ, Kim ST, Youn HD (2005) CtBP represses p300-mediated transcriptional activation by direct association with its bromodomain. Nat Struct Mol Biol 12: 423-428.
35. Shi Y, Sawada J, Sui G, Affar el B, Whetstine JR, et al. (2003) Coordinated histone modifications mediated by a CtBP co-repressor complex. Nature 422: 735-738.
36. Chinnadurai G (2002) CtBP, an unconventional transcriptional corepressor in development and oncogenesis. Mol Cell 9: 213-224.
37. Ohlsson R, Lobanenkov V, Klenova E Does CTCF mediate between nuclear organization and gene expression? Bioessays 32: 37-50.
38. Bogdanovic O, Veenstra GJ (2009) DNA methylation and methyl-CpG binding proteins: developmental requirements and function. Chromosoma 118: 549-565.
39. Adewumi O, Aflatoonian B, Ahrlund-Richter L, Amit M, Andrews PW, et al. (2007) Characterization of human embryonic stem cell lines by the International Stem Cell Initiative. Nat Biotechnol 25: 803-816.
40. Kouzarides T (2007) Chromatin modifications and their function. Cell 128: 693-705.
41. Zhou W, Zhu P, Wang J, Pascual G, Ohgi KA, et al. (2008) Histone H2A monoubiquitination represses transcription by inhibiting RNA polymerase II transcriptional elongation. Mol Cell 29: 69-80.
42. Choi HK, Kim BJ, Seo JH, Kang JS, Cho H, et al. (2010) Cohesion establishment factor, Eco1 represses transcription via association with histone demethylase, LSD1. Biochemical and biophysical research communications 394: 1063-1068.
43. Margueron R, Li G, Sarma K, Blais A, Zavadil J, et al. (2008) Ezh1 and Ezh2 maintain repressive chromatin through different mechanisms. Mol Cell 32: 503-518.
44. Shen X, Liu Y, Hsu YJ, Fujiwara Y, Kim J, et al. (2008) EZH1 mediates methylation on histone H3 lysine 27 and complements EZH2 in maintaining stem cell identity and executing pluripotency. Mol Cell 32: 491-502.
45. Sterner DE, Berger SL (2000) Acetylation of histones and transcription-related factors. Microbiol Mol Biol Rev 64: 435-459.
46. Gallinari P, Di Marco S, Jones P, Pallaoro M, Steinkuhler C (2007) HDACs, histone deacetylation and gene transcription: from molecular biology to cancer therapeutics. Cell Res 17: 195-211.
47. Hakimi MA, Bochar DA, Chenoweth J, Lane WS, Mandel G, et al. (2002) A core-BRAF35 complex containing histone deacetylase mediates repression of neuronal-specific genes. Proc Natl Acad Sci U S A 99: 7420-7425.
48. Soliman MA, Riabowol K (2007) After a decade of study-ING, a PHD for a versatile family of proteins. Trends Biochem Sci 32: 509-519.
49. Fazzio TG, Huff JT, Panning B (2008) Chromatin regulation Tip(60)s the balance in embryonic stem cell self-renewal. Cell Cycle 7: 3302-3306.
50. Unoki M, Kumamoto K, Takenoshita S, Harris CC (2009) Reviewing the current classification of inhibitor of growth family proteins. Cancer Sci 100: 1173-1179.

51. Nottke A, Colaiacovo MP, Shi Y (2009) Developmental roles of the histone lysine demethylases. Development 136: 879-889.
52. Wang J, Scully K, Zhu X, Cai L, Zhang J, et al. (2007) Opposing LSD1 complexes function in developmental gene activation and repression programmes. Nature 446: 882-887.
53. Fei T, Xia K, Li Z, Zhou B, Zhu S, et al. Genome-wide mapping of SMAD target genes reveals the role of BMP signaling in embryonic stem cell fate determination. Genome Res 20: 36-44.
54. Clague MJ, Coulson JM, Urbe S (2008) Deciphering histone 2A deubiquitination. Genome Biol 9: 202.
55. Avvakumov N, Cote J (2007) The MYST family of histone acetyltransferases and their intimate links to cancer. Oncogene 26: 5395-5407.
56. Nishihara E, Moriya T, Shinohara K (2007) Expression of steroid receptor coactivator-1 is elevated during neuronal differentiation of murine neural stem cells. Brain Res 1135: 22-30.
57. Tetel MJ (2009) Nuclear receptor coactivators: essential players for steroid hormone action in the brain and in behaviour. J Neuroendocrinol 21: 229-237.
58. Feige E, Motro B (2002) The related murine kinases, Nek6 and Nek7, display distinct patterns of expression. Mech Dev 110: 219-223.
59. Kreis P, Barnier JV (2009) PAK signalling in neuronal physiology. Cellular signalling 21: 384-393.
60. Thompson M (2009) Polybromo-1: the chromatin targeting subunit of the PBAF complex. Biochimie 91: 309-319.
61. Kinkley S, Staege H, Mohrmann G, Rohaly G, Schaub T, et al. (2009) SPOC1: a novel PHD-containing protein modulating chromatin structure and mitotic chromosome condensation. Journal of cell science 122: 2946-2956.
62. Shi YJ, Matson C, Lan F, Iwase S, Baba T, et al. (2005) Regulation of LSD1 histone demethylase activity by its associated factors. Mol Cell 19: 857-864.
63. Guccione E, Bassi C, Casadio F, Martinato F, Cesaroni M, et al. (2007) Methylation of histone H3R2 by PRMT6 and H3K4 by an MLL complex are mutually exclusive. Nature.
64. Isono K, Fujimura Y, Shinga J, Yamaki M, J OW, et al. (2005) Mammalian polyhomeotic homologues Phc2 and Phc1 act in synergy to mediate polycomb repression of Hox genes. Mol Cell Biol 25: 6694-6706.
65. Osley MA (2006) Regulation of histone H2A and H2B ubiquitylation. Brief Funct Genomic Proteomic 5: 179-189.
66. Sassone-Corsi P, Mizzen CA, Cheung P, Crosio C, Monaco L, et al. (1999) Requirement of Rsk-2 for epidermal growth factor-activated phosphorylation of histone H3. Science 285: 886-891.
67. Sun XJ, Wei J, Wu XY, Hu M, Wang L, et al. (2005) Identification and characterization of a novel human histone H3 lysine 36-specific methyltransferase. J Biol Chem 280: 35261-35271.
68. Huen MS, Sy SM, van Deursen JM, Chen J (2008) Direct interaction between SET8 and proliferating cell nuclear antigen couples H4-K20 methylation with DNA replication. J Biol Chem 283: 11073-11077.
69. Jiang Y, Jakovcevski M, Bharadwaj R, Connor C, Schroeder FA, et al. Setdb1 histone methyltransferase regulates mood-related behaviors and expression of the NMDA receptor subunit NR2B. J Neurosci 30: 7152-7167.
70. Ito S, D'Alessio AC, Taranova OV, Hong K, Sowers LC, et al. (2010) Role of Tet proteins in 5mC to 5hmC conversion, ES-cell self-renewal and inner cell mass specification. Nature 466: 1129-1133.
71. Hamamoto R, Furukawa Y, Morita M, Iimura Y, Silva FP, et al. (2004) SMYD3 encodes a histone methyltransferase involved in the proliferation of cancer cells. Nat Cell Biol 6: 731-740.
72. Cunliffe VT (2008) Eloquent silence: developmental functions of Class I histone deacetylases. Current opinion in genetics & development 18: 404-410.
73. Kim J, Guermah M, McGinty RK, Lee JS, Tang Z, et al. (2009) RAD6-Mediated transcription-coupled H2B ubiquitylation directly stimulates H3K4 methylation in human cells. Cell 137: 459-471.
74. Zhang XY, Varthi M, Sykes SM, Phillips C, Warzecha C, et al. (2008) The putative cancer stem cell marker USP22 is a subunit of the human SAGA complex required for activated transcription and cell-cycle progression. Mol Cell 29: 102-111.
75. Marango J, Shimoyama M, Nishio H, Meyer JA, Min DJ, et al. (2008) The MMSET protein is a histone methyltransferase with characteristics of a transcriptional corepressor. Blood 111: 3145-3154.
76. Nimura K, Ura K, Shiratori H, Ikawa M, Okabe M, et al. (2009) A histone H3 lysine 36 trimethyltransferase links Nkx2-5 to Wolf-Hirschhorn syndrome. Nature 460: 287-291.
