# Supplementary material for: Extensive Transcriptional Regulation of Chromatin Modifiers during Human Neurodevelopment
Source: PLoS One. 2012 May 9;7(5):e36708. doi: 10.1371/journal.pone.0036708 (PMC3348879; doi:10.1371/journal.pone.0036708)
Supplement: Figure S5 — Expression of epigenetic regulators normalized to hESC. hESC were differentiated into NEP or NCP. RNA was prepared from all types or cortical tissue samples. qPCR was performed using primers specific for the indicated neurodevelopmental regulator genes. Threshold cycle values (Ct) were measured with a Biorad light cycler. Ct values were normalized to house keeping genes, and relative gene expressions were calculated by normalization to hESC expression levels. Data are means of three independent differentiations +/− standart deviation (SD). p-values were calculated with Studens t-test and corrected for false discovery rate (FDR) according to Benjamini-Hochberg. They correspond to the statistical difference from the expression levels in hESC. Data corresponds to Fig. 2, 5, 6, and 7. (PDF) [file pone.0036708.s005.pdf]

Figure S5: Expression of epigenetic regulators normalized to hESC

Gene	rel. expr. NEP	SD	FDR corr. p-value	rel. expr. NCP	SD	FDR corr. p-value	rel. expr. Ctx	SD	FDR corr. p-value
ARID1A	3.1	0.4	0.022	1.1	0.6	0.912	1.4	1.0	0.709
ASH1L	3.2	0.6	0.022	3.2	0.9	0.031	13.3	4.5	0.015
ASXL1	13.8	0.8	0.000	7.1	2.7	0.032	24.8	5.2	0.008
ATF2	3.2	0.6	0.021	1.7	0.7	0.201	8.4	0.9	0.000
AURKA	1.2	0.1	0.159	0.5	0.2	0.073	0.5	0.1	0.051
AURKB	1.2	0.3	0.370	0.4	0.2	0.081	0.0	0.0	0.010
AURKC	2.4	0.9	0.075	0.4	0.2	0.090	1.3	0.7	0.614
BAF45A	2.5	1.5	0.106	1.6	0.9	0.404	13.5	4.7	0.010
BAF53A	1.1	0.6	0.939	0.8	0.4	0.438	19.2	10.9	0.024
BAF53B	7.6	9.1	0.155	5.3	4.2	0.341	576.7	331.6	0.013
BAF60A	1.5	0.6	0.291	1.0	0.5	0.695	2.6	0.6	0.029
BAF60C	5.1	1.9	0.036	10.8	8.3	0.042	495.4	116.2	0.010
BAZ1A	3.6	0.6	0.021	2.4	0.4	0.032	6.1	2.9	0.029
BAZ1B	2.1	0.5	0.039	1.3	0.0	0.187	1.3	0.7	0.640
BAZ2A	2.6	0.1	0.023	0.8	0.1	0.213	1.4	0.7	0.495
BAZ2B	5.0	1.5	0.022	1.7	0.9	0.325	11.3	5.8	0.024
BMI1	7.4	1.2	0.017	8.8	0.9	0.023	28.0	11.7	0.010
BPTF	3.0	1.0	0.050	1.2	0.5	0.759	4.4	3.1	0.054
BRD1	3.1	0.6	0.022	2.0	0.6	0.042	1.5	0.5	0.181
BRD2	1.4	0.2	0.116	0.8	0.1	0.177	1.3	0.3	0.285
BRD3	1.5	0.6	0.291	0.7	0.1	0.115	0.4	0.2	0.073
BRD4	2.4	0.7	0.070	1.0	0.2	0.951	1.0	0.1	0.936
BRD7	2.2	0.2	0.045	0.5	0.1	0.056	2.1	1.1	0.126
BRD8	2.4	0.4	0.025	1.0	0.3	0.962	3.5	1.2	0.023
BRDT	0.6	0.4	0.358	0.2	0.1	0.065	325.1	559.2	0.341
BRPF1	1.6	0.3	0.128	1.9	0.4	0.074	2.6	0.7	0.036
BRPF3	2.2	0.4	0.027	1.1	0.1	0.402	1.7	0.3	0.041
BRWD1	2.8	0.6	0.023	0.8	0.2	0.321	5.6	0.4	0.010
BRWD2	2.1	0.4	0.027	1.0	0.1	0.827	2.3	0.2	0.010
BRWD3	1.7	0.4	0.080	0.5	0.2	0.106	1.2	0.1	0.058
CARM1	1.2	0.3	0.295	1.1	0.1	0.391	0.4	0.0	0.008
CBX1	2.1	0.5	0.045	0.7	0.4	0.326	0.8	0.4	0.447
CBX3	2.1	0.6	0.063	1.1	0.1	0.219	0.6	0.1	0.035
CBX4	3.4	0.8	0.022	2.8	0.2	0.014	4.8	0.8	0.009
CBX5	3.1	0.4	0.024	1.5	0.3	0.092	1.0	0.4	0.844
CBX6	3.3	1.3	0.031	12.8	4.2	0.015	11.4	2.5	0.010
CBX7	1.8	0.1	0.021	0.6	0.4	0.276	61.0	5.7	0.007
CBX8	10.9	1.9	0.019	5.7	2.3	0.028	7.2	1.8	0.011
CDYL	1.1	0.2	0.909	0.4	0.0	0.019	0.6	0.2	0.115
CDYL2	5.9	3.4	0.066	0.5	0.1	0.032	19.4	8.9	0.020
CHD1	3.8	0.8	0.023	1.4	0.1	0.108	2.0	0.3	0.025
CHD2	3.8	1.3	0.040	1.3	0.2	0.187	3.9	1.5	0.033
CHD3	1.7	0.8	0.226	4.0	1.2	0.024	3.5	0.3	0.021
CHD4	3.4	1.3	0.044	1.2	0.2	0.511	1.9	1.2	0.317
CHD5	1.0	0.1	0.915	0.7	0.4	0.331	67.7	35.9	0.010
CHD6	4.0	0.7	0.023	2.2	0.9	0.096	3.7	2.2	0.051
CHD7	6.0	1.9	0.023	0.1	0.1	0.042	2.6	0.1	0.009

CHD8	1.9	0.6	0.053	0.7	0.1	0.156	2.1	0.6	0.043
CHD9	4.6	0.3	0.042	5.0	1.9	0.031	7.0	2.3	0.019
CIITA	4.8	4.6	0.174	10.8	7.5	0.058	15.2	4.1	0.029
CSRP2BP	1.5	0.2	0.033	1.0	0.3	0.819	1.3	0.6	0.634
CTBP1	2.8	1.0	0.048	1.5	0.0	0.046	3.5	0.4	0.008
CTBP2	1.2	0.1	0.157	1.2	0.2	0.398	1.4	0.6	0.323
CTCF	1.7	0.2	0.030	0.8	0.1	0.184	1.0	0.5	0.880
DNMT1	2.9	1.8	0.100	6.5	4.3	0.120	8.9	8.0	0.273
DNMT3A	1.6	0.3	0.047	0.8	0.3	0.346	0.3	0.1	0.042
DNMT3B	0.1	0.1	0.038	0.0	0.0	0.018	0.0	0.0	0.010
DOT1L	2.0	0.4	0.033	0.4	0.0	0.016	0.5	0.3	0.155
DZIP3	2.1	0.1	0.021	0.6	0.2	0.143	2.8	0.9	0.036
EED	3.4	0.5	0.022	1.0	0.2	0.940	2.5	1.0	0.053
EHMT2	2.0	0.3	0.030	0.6	0.2	0.078	1.0	0.1	0.478
ESCO1	1.3	0.2	0.108	0.6	0.2	0.131	1.8	0.6	0.121
ESCO2	1.7	0.5	0.121	2.0	0.6	0.061	0.1	0.0	0.010
EZH1	1.9	0.6	0.174	1.7	0.8	0.299	24.8	7.7	0.010
EZH2	3.3	0.5	0.044	1.0	0.2	0.905	0.1	0.1	0.016
HAT1	1.8	0.2	0.021	1.4	0.2	0.038	1.5	0.3	0.117
HDAC1	2.4	0.4	0.021	0.5	0.2	0.112	1.6	0.9	0.337
HDAC10	2.2	0.5	0.040	2.0	0.7	0.106	2.7	0.7	0.025
HDAC11	1.8	0.8	0.128	3.0	1.4	0.099	6.0	2.8	0.042
HDAC2	2.5	0.3	0.070	1.3	0.3	0.414	9.3	1.2	0.023
HDAC3	1.3	0.3	0.205	1.1	0.4	0.851	1.6	0.5	0.163
HDAC4	1.9	0.4	0.043	0.7	0.2	0.146	6.0	1.1	0.012
HDAC5	5.0	1.6	0.025	1.5	0.7	0.382	18.5	3.1	0.008
HDAC6	4.6	0.9	0.023	2.5	0.4	0.028	6.0	1.1	0.013
HDAC7	4.7	4.3	0.132	6.2	3.1	0.048	11.0	5.7	0.033
HDAC8	4.8	0.2	0.022	3.3	0.8	0.025	5.8	2.1	0.030
HDAC9	18.8	8.5	0.021	16.8	6.3	0.023	44.5	21.6	0.013
HINFP	1.7	0.1	0.229	0.6	0.2	0.199	1.2	0.1	0.556
ING1	2.0	0.6	0.103	0.9	0.2	0.618	2.4	0.5	0.042
ING2	1.8	0.4	0.038	1.1	0.2	0.401	2.1	0.6	0.036
ING3	3.3	0.3	0.027	1.5	0.3	0.127	3.1	1.1	0.029
ING4	3.8	0.2	0.020	4.2	1.9	0.059	3.1	0.5	0.012
ING5	2.3	0.3	0.021	0.9	0.2	0.477	2.2	0.5	0.026
INO80	1.5	0.2	0.040	1.0	0.1	0.929	2.1	0.5	0.029
KAT2A	2.1	0.7	0.074	1.1	0.1	0.410	3.3	1.2	0.045
KAT2B	21.6	9.5	0.020	13.9	8.0	0.031	235.0	97.3	0.008
KAT5	2.0	0.6	0.073	1.1	0.0	0.031	3.1	1.6	0.074
KDM1	1.9	0.2	0.024	0.6	0.1	0.047	0.6	0.2	0.131
KDM4A	0.9	0.2	0.665	0.7	0.1	0.092	0.8	0.2	0.244
KDM4C	1.4	0.1	0.040	0.4	0.1	0.024	2.2	0.4	0.017
KDM5B	1.3	0.2	0.048	0.6	0.3	0.188	0.4	0.3	0.104
KDM5C	1.5	0.2	0.263	1.2	0.5	0.796	0.7	0.5	0.386
KDM6B	3.2	0.6	0.021	1.3	0.5	0.350	1.1	0.1	0.460
MBD1	2.4	0.6	0.045	1.5	0.1	0.081	2.2	0.3	0.012
MBD2	3.3	0.4	0.018	1.9	0.6	0.054	4.1	0.6	0.009
MBD3	1.8	0.6	0.088	1.8	0.3	0.042	2.6	1.0	0.032
MBD4	2.4	0.4	0.023	0.5	0.1	0.020	2.6	0.6	0.029

MECP2	8.4	1.4	0.019	3.8	1.1	0.031	23.3	4.5	0.008
MLL	2.6	1.2	0.145	0.9	0.3	0.519	3.8	1.7	0.056
MLL3	2.9	1.4	0.103	1.9	0.8	0.158	3.7	1.6	0.047
MLL5	3.5	1.1	0.035	2.3	0.8	0.056	9.9	1.5	0.005
MTA1	1.6	0.5	0.151	0.7	0.0	0.131	0.7	0.2	0.155
MTA2	2.1	0.5	0.049	1.1	0.3	0.741	1.2	0.8	0.915
MYSM1	1.3	0.3	0.174	0.4	0.1	0.027	1.5	0.2	0.036
MYST1	2.4	0.3	0.019	1.3	0.4	0.545	2.8	0.6	0.015
MYST2	2.0	0.5	0.074	0.5	0.1	0.048	0.8	0.2	0.357
MYST3	5.5	1.6	0.021	3.6	1.1	0.030	14.6	9.8	0.029
MYST4	12.7	8.1	0.038	9.6	5.5	0.047	110.6	64.6	0.012
NAB2	5.5	3.9	0.062	4.5	1.2	0.020	1.5	0.2	0.067
NCOA1	3.1	0.9	0.044	5.4	2.3	0.032	39.1	33.3	0.016
NCOA3	3.8	0.6	0.019	1.2	0.5	0.684	2.5	0.5	0.015
NCOA6	9.0	0.8	0.019	5.7	0.8	0.016	20.1	5.0	0.010
NEK6	5.9	3.6	0.049	7.8	1.3	0.000	10.0	4.9	0.026
NSD1	2.0	0.4	0.079	0.7	0.1	0.114	2.3	0.3	0.010
PAK1	0.8	0.2	0.233	0.5	0.1	0.026	0.8	0.4	0.364
PBRM1	3.7	0.7	0.018	1.9	0.2	0.024	3.5	1.0	0.014
PCGF1	2.3	0.4	0.027	1.7	0.4	0.077	3.0	0.6	0.022
PCGF2	2.1	0.0	0.031	1.2	0.2	0.292	1.2	0.2	0.497
PCGF3	4.3	0.6	0.021	2.4	1.5	0.241	6.3	1.2	0.009
PCGF5	3.5	0.9	0.021	1.4	0.0	0.144	34.4	14.3	0.009
PCGF6	1.8	0.1	0.054	0.8	0.1	0.195	1.1	0.2	0.647
PHC1	0.2	0.0	0.023	0.1	0.0	0.012	0.1	0.1	0.029
PHC2	26.9	9.9	0.018	18.0	4.3	0.012	11.8	2.7	0.011
PHF1	4.5	1.2	0.098	1.7	0.4	0.433	12.5	6.5	0.029
PHF13	2.2	1.2	0.133	1.1	0.3	0.815	0.5	0.2	0.074
PHF2	6.2	0.5	0.021	7.0	2.2	0.020	12.7	4.7	0.010
PHF21A	3.4	1.4	0.045	1.6	0.6	0.265	4.9	2.0	0.025
PHF21B	3.5	1.2	0.030	0.3	0.2	0.091	6.2	6.2	0.129
PHF3	2.2	0.6	0.039	1.0	0.2	0.826	2.0	0.8	0.129
PHF5A	1.7	0.4	0.104	0.5	0.1	0.069	0.9	0.0	0.453
PHF6	4.0	1.1	0.021	0.6	0.3	0.246	1.3	0.2	0.228
PHF7	1.1	0.2	0.808	0.6	0.3	0.205	1.3	0.2	0.346
PRMT1	1.4	0.4	0.141	0.6	0.0	0.036	0.4	0.0	0.021
PRMT2	2.5	1.0	0.056	2.8	0.4	0.025	4.6	1.4	0.023
PRMT3	1.6	0.2	0.106	0.6	0.1	0.082	1.6	0.9	0.497
PRMT5	1.3	0.1	0.164	0.7	0.1	0.110	0.9	0.5	0.588
PRMT6	1.7	0.6	0.153	2.1	0.1	0.032	1.4	1.1	0.841
PRMT7	1.8	0.1	0.021	1.5	0.5	0.191	4.1	0.9	0.016
PRMT8	1.4	0.5	0.237	5.7	3.5	0.077	20.5	15.0	0.028
RING1	4.3	0.4	0.023	2.7	0.7	0.032	8.4	3.6	0.013
RNF2	2.8	0.4	0.023	1.4	0.2	0.085	0.9	0.2	0.406
RNF20	1.6	0.2	0.031	1.3	0.1	0.053	3.5	1.6	0.044
RPS6KA3	7.2	0.1	0.021	7.0	0.6	0.025	15.6	5.2	0.011
RPS6KA5	4.8	1.8	0.038	0.8	0.5	0.476	21.6	6.6	0.008
SETD1A	1.7	0.5	0.092	1.2	0.2	0.242	1.3	0.2	0.107
SETD1B	1.9	0.5	0.072	1.8	0.4	0.041	2.5	0.5	0.016
SETD2	2.8	0.4	0.019	1.9	0.5	0.060	4.8	2.4	0.035

SETD3	1.5	0.2	0.048	1.0	0.3	0.947	4.2	2.1	0.042
SETD4	2.1	0.3	0.049	0.9	0.1	0.609	3.1	1.0	0.029
SETD5	2.3	0.4	0.022	1.3	0.3	0.237	0.8	0.2	0.256
SETD6	2.6	0.4	0.021	0.6	0.2	0.136	2.0	0.5	0.068
SETD7	8.2	0.5	0.025	10.5	1.5	0.020	48.7	14.5	0.007
SETD8	3.0	0.6	0.023	3.0	0.7	0.030	4.3	1.5	0.028
SETDB1	2.5	0.7	0.038	1.1	0.2	0.801	1.2	0.2	0.307
SETDB2	2.4	0.1	0.022	1.6	0.3	0.045	5.6	1.2	0.010
SMARCA2	0.7	0.3	0.333	29.5	9.7	0.035	63.9	19.1	0.006
SMARCA4	2.2	0.9	0.131	0.5	0.0	0.105	1.1	0.3	0.776
SMYD3	2.0	0.2	0.053	0.9	0.1	0.555	1.9	1.3	0.317
SPEN	3.5	1.4	0.037	1.0	0.3	0.982	4.8	2.2	0.024
SUV39H1	1.1	0.3	0.641	0.6	0.1	0.033	1.9	0.6	0.100
SUV420H1	2.4	0.9	0.049	1.2	0.3	0.533	3.0	0.7	0.024
SUZ12	2.7	0.6	0.023	1.1	0.2	0.784	1.2	0.1	0.235
TET1	1.1	0.5	0.890	0.6	0.5	0.290	0.6	0.3	0.304
TET2	5.5	2.0	0.025	4.4	2.7	0.076	56.5	0.9	0.010
TRIM27	3.0	0.4	0.021	1.7	0.6	0.166	2.6	1.4	0.105
UBE2A	2.5	0.5	0.038	3.2	0.5	0.042	7.2	3.2	0.017
UBE2B	2.3	0.2	0.038	2.7	0.3	0.032	6.0	1.4	0.010
USP16	2.6	0.2	0.020	3.3	0.4	0.013	9.1	3.7	0.023
USP21	2.4	0.4	0.027	2.2	0.5	0.041	7.2	2.7	0.024
USP22	1.6	0.5	0.134	1.2	0.2	0.190	1.2	0.3	0.291
WHSC1	4.3	0.5	0.019	1.3	0.1	0.030	2.3	1.1	0.105
ZMYND8	1.0	0.2	0.883	0.4	0.1	0.034	2.0	1.0	0.186
