# Supplementary figures and images for: Extensive Transcriptional Regulation of Chromatin Modifiers during Human Neurodevelopment
Source: PLoS One. 2012 May 9;7(5):e36708. doi: 10.1371/journal.pone.0036708 (PMC3348879; doi:10.1371/journal.pone.0036708)

Figure S6: Comparison of relative expression data from microarray and qPCR

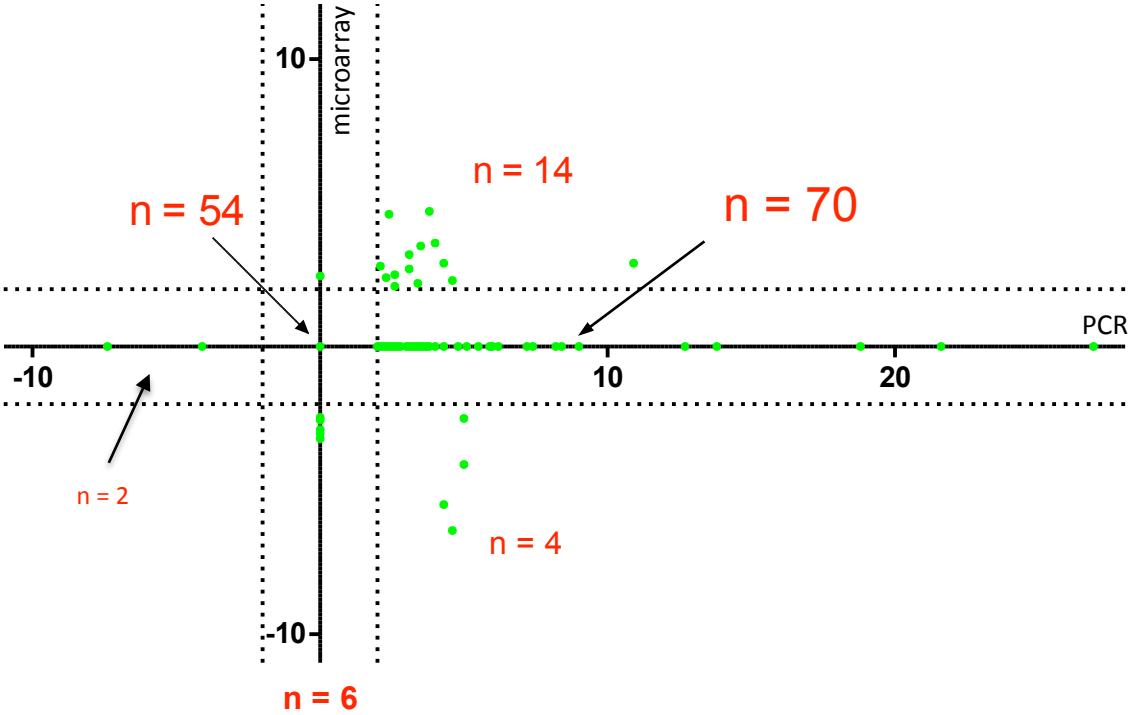

Supplement: Figure S6 — Comparison of relative expression data from microarray and qPCR. hESC were differentiated into NEP, and RNA was prepared from undifferentiated hESC and NEP. qPCR was performed using primers specific for the 150 epigenetic regulator genes and threshold cycle values (Ct) were measured with a Biorad light cycler. Ct values of NEP were first normalized to house keeping genes. Fold expression levels were obtained by further normalization to hESC (Fig. S5). RNA for microarray was prepared as described above and hybridization on Affymetrix gene chips was perfomed. After bioinformatic analysis, we obtained expression levels relative to hESC. Microarray data were screened for our set of 150 epigenetic regulator genes investigated by qPCR. Fold expression values obtained from microarray data are blotted on the y-axis, and fold expression levels obtained from qPCR are blotted on the x-axis. For both data sets, non- significant values were set to 0. n: amount of genes in the different groups. Dotted lines indicate 2-fold regulations. (PDF) [file pone.0036708.s006.pdf]
